# Association of single nucleotide polymorphism at long non-coding RNA 8138.1 with duration of fertility in egg-laying hens

**DOI:** 10.7717/peerj.7282

**Published:** 2019-07-12

**Authors:** Adeyinka Abiola Adetula, Syed Ali Azmal, Chenghao Sun, Abdelmotaleb Elokil, Shijun Li

**Affiliations:** 1 Key Laboratory of Agricultural Animal Genetics, Breeding, and Reproduction of the Ministry of Education, Huazhong Agricultural University, Wuhan, Hubei, China; 2 Department of Livestock Services (DLS), Under the Ministry of Fisheries and Livestock (MOFL), Dhaka, Bangladesh; 3 Huadu Yukou Poultry Industry Co. Ltd, Beijing, Hubei, China; 4 Department of Animal Production, Faculty of Agriculture, Benha University, Moshtohor, Egypt

**Keywords:** Egg-laying hens, Long non-coding RNA, SNP, Genotypes, Association analysis

## Abstract

A previous genome-wide transcriptional analysis identified long non-coding RNA 8138.1 (*lncRNA8138.1*) as a candidate gene related to hen duration of the fertility (DF) trait. *LncRNA8138.1* gene response to growth factor and reproductive system development suggests it has a vital role in reproduction. In this study, we investigated the *lncRNA8138.1* gene sequence in a population of egg-laying hens. The sequence analysis of the *lncRNA8138.1* gene containing about 1.6 k nucleotides (nt) was observed with four single nucleotide polymorphisms (SNPs) and 7 nt indel including r.4937159A > G; r.4937219T > C; r.4937258G > C; r.4937318C > G and g.4937319_4937325delinsTGTGTGG. Next, the genomic DNAs from laying hen populations were subjected to polymerase chain reaction (PCR) and restriction fragment length polymorphism (RFLP) to detect a region of 457 bp carrying *lncRNA8138.1* r.4937159A > G substitution. Further inspection of the region containing r.4937159A > G mutation revealed three genotypes viz., AA, AG, and GG were observed with respective frequencies of 0.106, 0.607, and 0.287 in laying hen population 1 (P1) (*n* = 1, 042) and respective frequencies of 0.176, 0.708, and 0.116 in laying hen population 2 (P2) (*n* = 826). Moreover, to further examining the frequencies of r.4937159A > G genotypes in P1 and P2, and their additive and dominance effects; r.4937159A > G locus was significantly associated with DF-trait in both P1 and P2 (EN: the number of eggs, FN: the number of fertile eggs after a single AI), and DN (the number of days post-insemination until last fertile egg). In testing for additive and dominance effects, additive effect was significant (*P* < 0.05) in both P1 and P2 for DF-trait, and the dominance effect was significant (*P* < 0.05) for EN and FN traits, suggesting that r.4937159A > G polymorphism is a potential biomarker for DF-trait. However, the identified novel r.4937159A > G mutation and others require further investigation to confirm phenotypic causality and potential genetic relationships with reproductive traits. Overall, our findings suggest the significance of genetic variation in long non-coding RNAs may assist in future breeding programs to improve selection for prolonged DF-trait.

## Introduction

Identification of genes and their association with economic traits in farm animals could assist in the genetic selection of breeding stock. In poultry production, duration of fertility (DF) is one of the most important traits defined by the number of day’s post-fertilisation when viable eggs are produced. In the past decade, scientific advances have fueled progress in poultry industry to identify several proteins and enzyme-coding genes that are associated with period of fertility in laying birds, including, carbonic anhydrase (Holm, Ridderstråle & Knutsson, 1996), avidin and avidin-related protein-2 (AVR2) (Daryabari et al., 2014) aquaporins (Bakst, 2011; Zaniboni & Bakst, 2004), alkaline phosphatase (Bakst & Akuffo, 2007), the progesterone receptor (Yoshimura, Koike & Okamoto, 2000), the transforming growth factor-β (*TGF-*β**) and its receptors (Das et al., 2006). These genes mostly affect immune system activity and metabolic processes implicated in maintaining sperm storage at the time of fertilization (Bakst, Wishart & Brillard, 1994). Hens often have prolonged DF, large and fertile egg production while some hens have short DF, with small and infertile eggs during the reproductive season. Both genetic as well as non-genetic causes have been described for DF trait in hens (Bakst, 2011; Holt & Fazeli, 2016; Sasanami et al., 2013).

The nuclei of avian cells composed of distinct molecules and transcriptome analysis indicate a major portion of the genome termed the non-coding RNAs (ncRNAs) which are generally classified into short and long non-coding RNAs (lncRNAs) depending on the length of the RNAs (Brosnan & Voinnet, 2009; Wang et al., 2017). Long non-coding RNAs (lncRNAs) have emerged as important molecules which underlie developmental and reproductive processes. LncRNAs required for gonadogenesis (Mulvey et al., 2014; Roeszler et al., 2012), sex determination (Zhang et al., 2010), sex hormone responses (Li et al., 2013; Yang et al., 2013), meiosis (Shichino, Yamashita & Yamamoto, 2014), spermatogenesis (Anguera et al., 2011; Arun et al., 2012; Wen et al., 2016), and Oogenesis (Jenny et al., 2006) have been identified in different organisms. Given the diverse roles, lncRNAs play in essential biological processes; however, the role of lncRNAs in regulating DF-trait has not been extensively studied, especially in egg-laying hens. Although large-scale RNA-sequencing data have revealed a large number of lncRNAs linked to special phenotypes in chickens (Adetula et al., 2018; Liu et al., 2017), none of these lncRNAs has been screened in a population of chickens for analyses of significant trait-associated single-nucleotide polymorphisms (SNPs). Currently, SNPs in lncRNA genes are one of the most common genetic variants of concern (Minotti et al., 2018); SNPs in lncRNA genes have been anticipated to contain some risk variants. For instance, emerging evidence has indicated the important role of genetic variants of lncRNAs in diseases (Jin et al., 2011; Tarighi et al., 2018) and chicken performance traits (Ren et al., 2017).

In 2018, a study applied a genome-guided strategy to reconstruct the uterovaginal junction transcriptome of egg-laying hens with long- and short-DF; and sought to uncover key lncRNA genes related to DF-trait by RNA-sequencing technology (Adetula et al., 2018). This study identified *lncRNA8138.1* gene function in the biological processes that can affect response to growth factor, reproductive system development, positive regulation of cell differentiation, developmental process involved in reproduction, regulation of osteoblast differentiation, and carbohydrate binding. Importantly, *lncRNA8138.1* gene was significantly differentially expressed between two groups (long- and short-DF hens), and its differential expression was confirmed by quantitative real-time polymerase chain reaction, suggesting that *lncRNA8138.1* is vital for animal reproduction. To date, the chicken *lncRNA8138.1* gene DNA polymorphisms are largely unexplored. Therefore, in this study, the SNP variants in *lncRNA8138.1* gene identified and the relationship between *lncRNA8138.1* mutation and DF-trait evaluated in large egg-laying hen populations. Our discovery provides a basis for further research about the underlying molecular markers that may help to improve the reproductive performance of hens.

## Materials & Methods

### Ethics statement

The protocols for all animal experiments were approved by the Scientific Ethic Committee of Huazhong Agricultural University with approval number HZAUCH-2018-005.

### Egg-laying hens and duration of fertility trait

Commercial Jing-Hong laying hen populations were utilised in this study. Laying hen population 1 (P1) was obtained from the poultry farm of Huadu Yukou Poultry Industry, Co. Ltd, Beijing, China and population 2 (P2) from Jingzhou Yukou Poultry Industry, Co Ltd, Jingzhou, China. Briefly, the egg-laying hen populations were from two elite breeding lines, line 1 (white egg) and line 2 (brown egg). All hens were kept under standard conditions from 25 weeks aiming to study their duration of fertility. Hens were inseminated once with 2 ×10^8^ pooled sperms ejaculates collected from viable rooster flocks. Eggs were collected and marked daily from day 2–20 after artificial insemination (AI); all hens completed a reproductive phase in three replicates and lasted 60 days. The number of egg per hen over the period was recorded, and the fertilized eggs were examined by candling on day 10 of incubation (dead embryos were considered as fertile). The reproductive history of all hens was recorded daily based on DF-trait: EN (number of eggs), FN (the number of fertile eggs after a single AI), and DN (the number of days post-insemination until last fertile egg). At the end of the reproductive season, a total of 1,868 hens had a record of EN, FN, and DN respectively.

### DNA extraction and quality assessment

For DNA experiments, genomic DNA was isolated from 0.5 ml blood obtained from the egg-laying hen populations (P1, *n* = 1,042 and P2, *n* = 826) by phenol-chloroform extraction method (Sambrook & Russell, 2006). The quality of genomic DNA samples was rated using Nanodrop 2000 Spectrophotometer (Thermo Scientific™ ND2000USCAN; Waltham, MA USA). DNA samples were diluted to a working concentration of 50 ng/µL and stored at 20 °C for further analysis.

### Primer design, PCR amplification, and SNP detection

*LncRNA8138.1* is a double-exon and a single-intron gene at chromosome 18: (4936949–4937389, 4937470–4938607 bp) (GenBank accession number: MK336169). Primer pairs (AACCTGAGCTTTCAACAGAC & CCCAACTGCTCCAACATTAG) were designed using Primer Premier Software version 5.0 for amplification of *lncRNA8138.1*. Assays were performed by PCR in DNA samples of 60 hens selected randomly to construct a DNA pool with equal DNA concentration of 50 ng/µl for each hen. PCR amplifications were performed in a 25 µl volume containing 50 ng pooled DNA, 2.5 µl of 10 × PCR buffer, 5 mM of dNTPs, 10 pmol of forward and reverse primer, 0.625U Taq DNA polymerase (Takara Biomedical Technology Co., Ltd. Beijing, China) and ddH2O. The reactions were performed under the following conditions: an initial pre-denaturing at 95 °C for 5 min; 35 cycles of 95 °C (20 s), 55 °C–60 °C (20 s) 72 °C (20 s); and a terminal incubation step at 72 °C for 5min. The PCR products were sequenced directly with Applied Biosystems 3730xl DNA Analyzer (Applied Biosystems^®^, Waltham, MA USA), and SNP discovery was conducted by the Seqman program of DNASTAR 7.1.0 software (DNASTAR, Inc., Madison, WI, USA).

### PCR amplification and polymorphism genotyping

The region containing the *lncRNA8138.1* polymorphism was amplified in a single PCR system with the necessary reagents, and the reaction conditions were identical to those described above. Genotyping of PCR products was performed by PCR-RFLP assay in both P1 and P2 individuals using the primer pairs (AGTCACAGACCAGTAGTTTT & CCTCTAAAA

TCTTAGCAGCA). The PCR-RFLP condition for amplifying was pre-denaturing at 95 °C for 3 min, and 32 cycles of denaturation at 95 °C for 30 s, annealing at 64 °C for 30 s, extension at 72 °C for 20 s, and elongation at 72 °C for 7 min (Adeyinka et al., 2017). Next, PCR-RFLP products were digested with an enzyme (*Hind III*) and electrophoresed using 3% agarose gel.

### Mutational analysis and structure prediction

Predicting the common secondary structure of lncRNA is an important step towards understanding the function (Blythe, Fox & Bond, 2016). Here, we experimentally predict the secondary structure of *lncRNA8138.1* r.4937159A>G polymorphism in chicken, which may be used to upgrade sequence/structure alignments for other species. DNA samples of hens were selected randomly to construct a DNA pool with equal DNA concentration of 50 ng/µl for 60 individuals having AA and GG genotypes. PCR amplification reactions were performed in a 20 µl volume containing 50 ng pooled DNA, 2.5 µl of 10 × PCR buffer, 5 mM of dNTPs, 10 pmol of forward and reverse primer, 0.625U Taq DNA polymerase (Takara Biomedical Technology Co., Ltd. Beijing, China) and ddH2O. The reactions were conducted under the following conditions: an initial pre-denaturing at 95 °C for 5 min; 35 cycles of 95 °C (20 s), 55 °C–60 °C (20 s) 72°C (20 s); and a terminal incubation step at 72 °C for 5min. The PCR products were sequenced with ABI3730xl DNA analyzer (Applied Biosystems^®^, Waltham, MA, USA). The resulting nucleotides from the genome of AA and GG genotypes were used to predict the common secondary structure of *lncRNA8138.1* in chicken with the use of web-based structure prediction programs known as Dynalign (http://rna.urmc.rochester.edu/RNAstructureWeb/Servers/dynalign/dynalign.html) (Reuter & Mathews, 2010).

### Statistical Analysis

Descriptive statistics for the DF-trait were calculated using an online based descriptive statistics calculator. The SNP genetic parameters including homozygosity (Ho), heterozygosity (He; Ho + He = 1) and polymorphism information content (PIC) were estimated according to methods developed by (Nei & Roychoudhury, 1974). PIC is an indicator of polymorphism that can be used to estimate genetic diversity. According to PIC values, the genetic variations were classified as high PIC (>0.5), medium (0.25 < PIC < 0.5) and low (PIC < 0.25) (Botstein et al., 1980). Genotypes were tested for Hardy-Weinberg equilibrium using the Chi-squared tests (R function HWE.chisq) implemented in the R environment (v 3.0.2) (Pouresmaeili et al., 2013). The statistical significance level was set at *P* <0.05. The relationship between *lncRNA8138.1* polymorphism and DF-trait in P1 and P2 were estimated based on the general linear model procedure of SAS v. 9.2 (SAS Institute Inc., Cary, NC, USA) (Littell, Stroup & Freund, 2002). Briefly, the following linear model was used:

\begin{eqnarray*}{Y}_{\mathrm{ijklm}}= \mathrm{\mu }+{B}_{\mathrm{i}}+{G}_{\mathrm{j}}+{A}_{\mathrm{k}}+{L}_{\mathrm{l}}+{e}_{\mathrm{ijklm}} \end{eqnarray*}

where: *Y*_ijklm_ represents the dependent variable (DF-trait), µ is the overall population means, *B*_i_ is the fixed effect of ith breed (*i* = 1), *G*_j_ is the fixed effect of jth genotype (j = AA, AG, and GG), *A*_k_ = is the fixed effect of kth age (*k* = 1), *L*_l_ is the fixed effect of lth location associated with the DF-trait, *e*_ijklm_ is the random error. Differences in means were considered significant at *P* <0.05 and the SAS PROC *t*-test (SAS v. 9.2; SAS Institute Inc., Cary, NC, USA) was used to estimate the differences in the LS Means between genotypes. Additive and dominance effects were estimated using REG procedure in (SAS v. 9.2 (SAS Institute Inc., Cary, NC, USA)). Statistically significant differences in the investigated populations for additive and dominance effects were estimated as a *t*-test for LS Means.

## Results

### Descriptive statistics of laying hens duration of fertility

**Figure 1:**
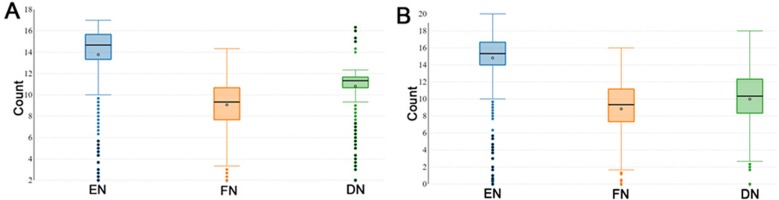
Boxplots showing duration of fertility trait. The boxplots show medians and interquartile ranges (IQRs) in (A) population 1 and (B) population 2. EN (number of eggs); FN (the number of fertile eggs after a single AI); and DN (the number of days post-insemination until last fertile egg).

During the reproductive season, DF-trait was defined by the EN (number of eggs), FN (the number of fertile eggs after a single AI) and DN (the number of days post-insemination until last fertile egg). The descriptive statistics of the recorded DF-trait in P1 (*n* = 1, 042) are shown in Fig. 1A. In the P1, the median EN, FN and DN were 14.67, 9.33 and 11.33 respectively and interquartile range (IQR) for the EN, FN, and DN were (13.33–15.67 eggs), (7.67–10.67 eggs) and DN, (10.67–11.67 days) respectively (Fig. 1A). Figure 1B illustrates the descriptive statistics of the recorded DF trait in the P2 (*n* = 826); the median EN, FN and DN were 15.33, 9.33 and 10.33, respectively. The IQR for the EN, FN and DN were (14.00–20.00 eggs), (7.33–11.17 eggs), and (8.33–12.33 days) respectively (Fig. 1B). In general, DF-trait showed high individual variability in both P1 and P2, even when we observed the same and optimal multiple insemination conditions.

### *LncRNA8138.1* genetic polymorphisms

**Figure 2:**
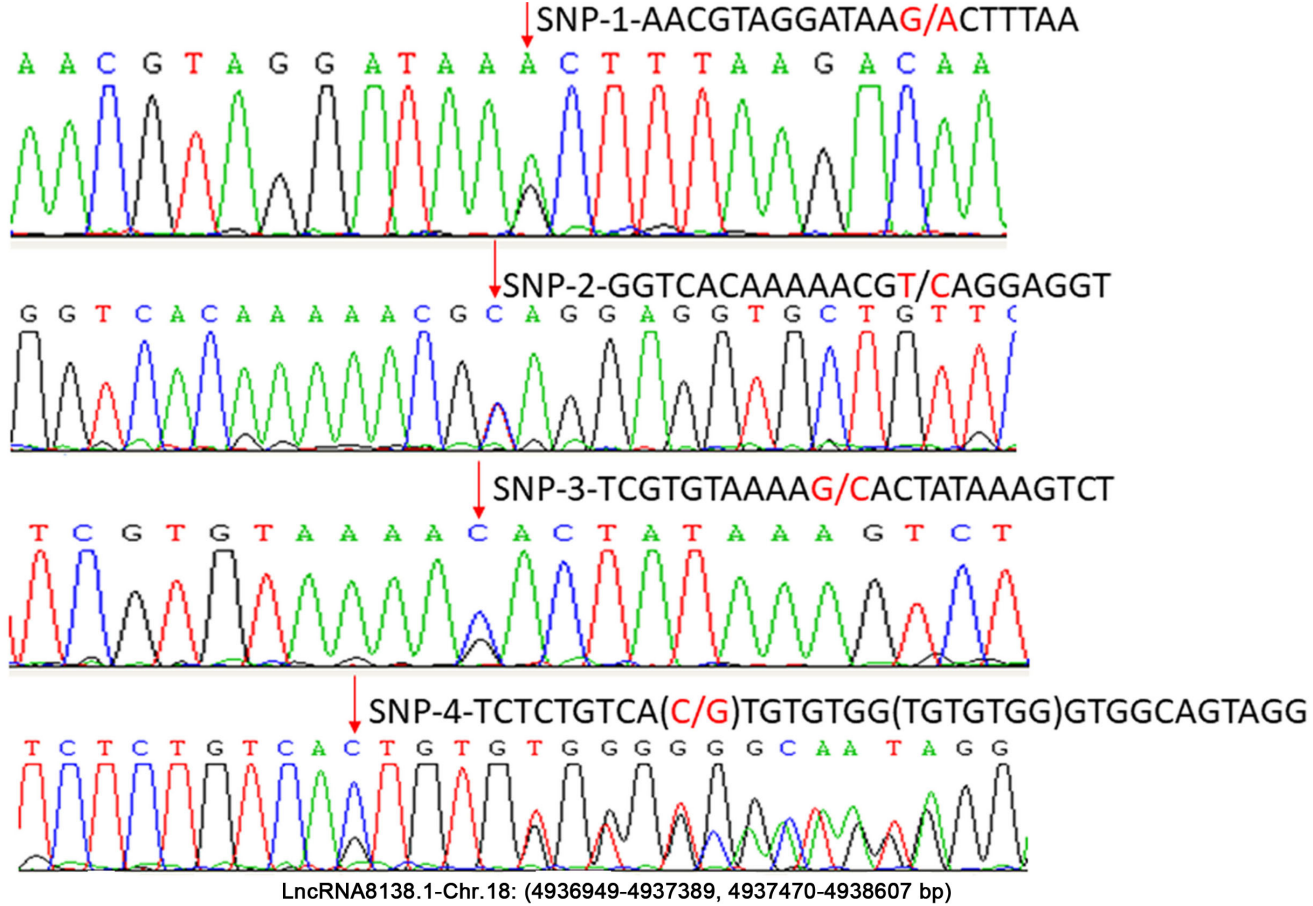
Sequence diagrams of chicken *lncRNA8138.1* gene SNP loci.

In this study, approximately 1.6 kb nucleotides of *lncRNA8138.1* were analysed. The analyses revealed a set of four possible SNP candidates and 7 nt indel in egg-laying hens that were detected through sequencing, including SNP r.4937159A>G; r.4937219T>C; r.4937258G>C; r.4937318C>G and g.4937319_4937325delinsTGTGTGG (Fig. 2). Several previous studies have reported that variants associated with lncRNA cannot be undervalued (Bhartiya & Scaria, 2016; Hrdlickova et al., 2014). Therefore, r.4937159A>G was detected by PCR-RFLP; the relationship with chicken DF-trait was further investigated in large egg-laying hen populations.

### Genetic parameters of the detected polymorphism

Table 1 showed the genotype frequency of *lncRNA8138.1* r.4937159A>G polymorphism in egg-laying hen populations. The *χ*^2^ test indicated that r.4937159A>G genotype frequency was not in agreement with HWE (*P* <0.05) in both P1 and P2 (Table 1). In the P1, data collected for r.4937159A>G polymorphism showed that “G” allele (0.591) was more frequent than “A” allele (0.409); and “G” allele (0.530) was often seen compared to “A” allele (0.470) in P2. The genotype frequencies of the majority of hens in P1 were either heterozygous AG (0.607), while (0.106) were homozygous AA and (0.287) were homozygous GG. In the P2, the majority were also heterozygous AG (0.708), (0.176) were homozygous AA, and (0.116) were homozygous GG (Table 1). In addition, polymorphism information content values revealed the genetic diversity of *lncRNA8138.1* r.4937159A>G polymorphism (PIC = 0.366 and 0.374) in P1 and P2, respectively (Table 1).

**Table 1:** Genotype and allele frequencies of lncRNA8138.1 (A/G) polymorphism in an egg-laying hen’s population.

Frequencies								
**Locus (r.4937159A>G)**	**Genotypes (numbers)**			**Alleles**	**Ho**	**He**	**PIC**	*χ*^2^ (*P*-value)^*^
P1: (*n* = 1,042)	AA (110)	AG (633)	GG (299)	0.409 (A)	0.516	0.483	0.366	68.450 (1.3E16)
	0.106	0.607	0.287	0.591(G)				
P2: (*n* = 826)	AA (145)	AG (585)	GG (96)	0.470 (A)	0.502	0.498	0.374	146.726 (9.01E34)
	0.176	0.708	0.116	0.530 (G)				

**Notes.**

* *P* value was computed by *χ*^2^ test.

### Common secondary structures of *lncRNA8138.1* mutation in chicken

**Figure 3:**
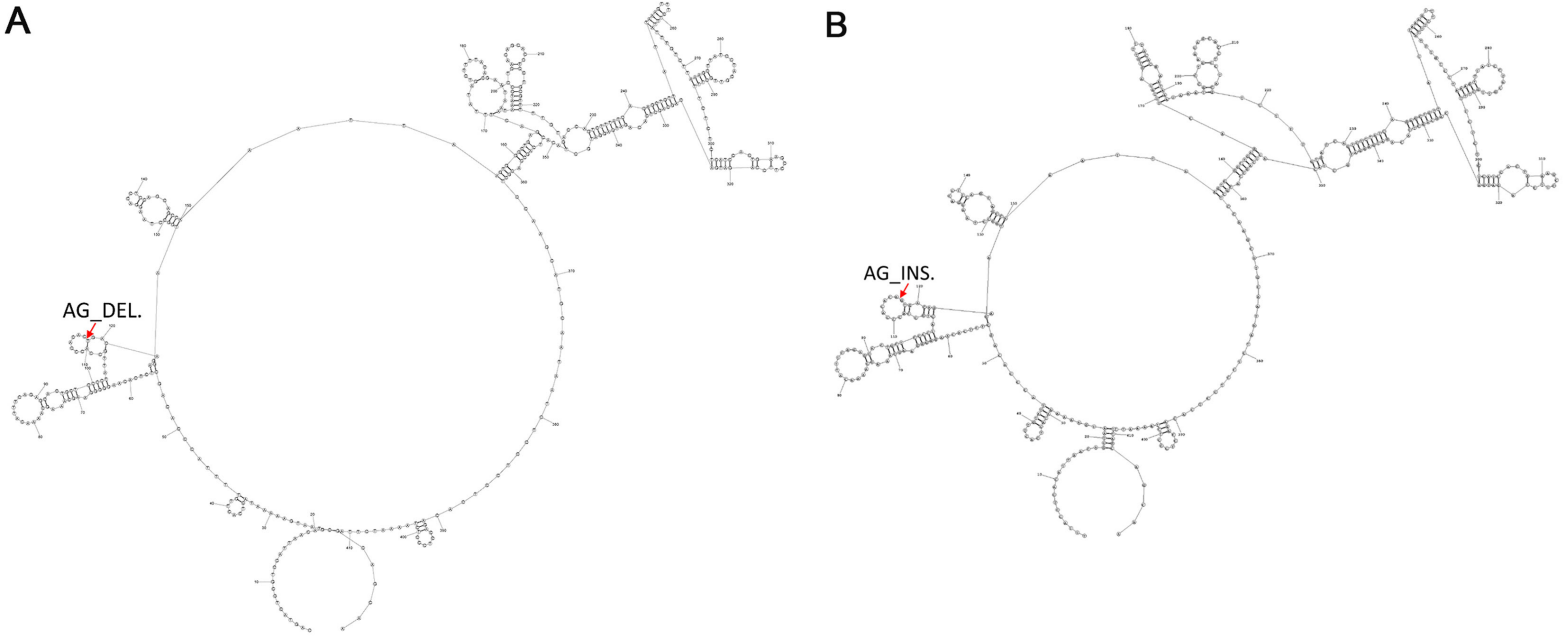
The 2-D map of a small region from the 5′ end of *lncRNA8138.1* gene showing common secondary structure elements. (A) The red arrow indicates the region with 2-point mutation (AG_deletion) in AA carriers and (B) the red arrow indicates the region with 2-point mutation (AG_insertion) in GG carriers. Common secondary structures were predicted by RNAstructure software version 6.1.

To determine the common secondary structures of *lncRNA8138.1* in chicken, the mutational profile of *lncRNA8138.1* gene was utilized for the structure prediction. This is done over individuals having AA and GG genotypes separately, and the results showed that individuals with AA genotype differed in secondary structure by 2-point mutations when compared with the individuals with GG genotype. The 2-point mutations represent the AG_deletion in the AA individuals (Fig. 3A) and AG_insertion in the GG individuals (Fig. 3B); however, a consistent pattern of similarity was noticed in both AA and GG genotypes secondary structures (Fig. 3).

### Analyses of associations between *lncRNA8138.1* nucleotide variation and DF trait

Associations between *lncRNA8138.1* r.4937159A>G polymorphism and DF-trait were investigated in the egg-laying hen populations. For all DF-trait (EN, FN and DN), LS Means and standard error of mean (SEM) values were tabulated for the genotype effect (Table 2). In the P1, association analysis confirmed that SNP r.4937159A>G was significantly related to DF-trait (EN: *P* < 0.0001, FN: *P* < 0.0001, and DN: *P* ≤ 0.05; Table 2). In the P2, a significant association was observed for the EN (*P* < 0.0001) and FN, (*P* < 0.01); Table 2). Besides, additive and dominance effects were significant for all DF-trait except DN in the P1 (*P* < 0.05, Table 2), whereas in the P2, additive effects were significant for EN and FN traits (*P* < 0.01, Table 2). Additionally, comparison of *lncRNA8138.1* r.4937159A>G genotypes revealed significant differences between genotypes, i.e., (GG vs AA, GG vs AG, and AA vs AG) respectively (Table 2).

**Table 2:** Association of *lncRNA8138.1* (A/G) polymorphism with DF-trait in hens.

**SNP**	**DF-Traits**	**Least-squares mean ± SEM**			***P*-value**	**Effect (µ ± SE)**				**Multiple comparison test *P*-value**		
		AA	AG	GG		Additive	*P*-value	Dominance	*P*-value	GG-AA	GG-AG	AA-AG
P1: r.4937159A>G		(*n* = 110)	(*n* = 633)	(*n* = 299)								
	^a^EN (eggs)	12.994 ± 0.291	13.562 ± 0.121	14.506 ± 0.176	<0.0001	−0.814 ± 0.158	<0.0001	0.269 ± 0.097	0.006	<0.0001	<0.0001	0.0717
	^b^FN (eggs)	8.567 ± 0.247	8.909 ± 0.103	9.575 ± 0.149	<0.0001	−0.554 ± 0.134	<0.0001	0.197 ± 0.083	0.017	0.001	0.000	0.201
	^c^DN (days)	10.638 ± 0.184	10.745 ± 0.077	11.048 ± 0.111	0.0474	−0.218 ± 0.098	0.027	0.087 ± 0.060	0.152	0.057	0.025	0.595
P2: r.4937159A>G		(*n* = 145)	(*n* = 585)	(*n* = 96)								
	^a^EN (eggs)	13.972 ± 0.276	14.719 ± 0.137	16.649 ± 0.339	<0.0001	−0.031 ± 0.005	<0.0001	0.006 ± 0.005	0.221	<0.0001	<0.0001	0.016
	^b^FN (eggs)	7.929 ± 0.277	8.948 ± 0.138	9.442 ± 0.339	0.001	0.020 ± 0.006	0.000	−0.015 ± 0.009	0.105	0.001	0.178	0.001
	^c^DN (days)	9.616 ± 0.281	9.982 ± 0.139	10.434 ± 0.345	0.183	−0.010 ± 0.006	0.066	−0.001 ± 0.005	0.878	0.066	0.225	0.244

**Notes.**

Data are summarized as means ± SEM. For all DF-traits, we examined multiple inseminations (up to about 60 wks. of age); (*n* = 1, 042; P1) and (*n* = 826; P2). DF (duration of fertility), ^*a*^EN (number of eggs); ^*b*^FN (the number of fertile eggs after a single AI); ^*c*^DN (the number of days post-insemination until last fertile egg). Genotypes comparisons p-values (GG vs AA, GG vs AG, and AA vs AG) were obtained from the genome of egg-laying hen populations.

## Discussion

Previously, Adetula et al. (2018) identified a novel *lncRNA8138.1* gene is closely related to DF-trait in egg-laying hens and several studies have also explored the role of lncRNAs in reproductive biology (Taylor et al., 2015) and their findings suggest that lncRNA gene has an essential role in reproduction. However, there are no previous reports of chicken *lncRNA8138.1* genetic variant and its relationship with DF-trait. The relationship between *lncRNA8138.1* gene variants and DF-trait in large egg-laying hen populations required further analysis. First, we measured the expression profiles of the chicken *lncRNA8138.1* gene in our previous study, and the results demonstrated that *lncRNA8138.1* was differentially expressed between long- and short-DF hens (Adetula et al., 2018). Given the numerous lncRNAs that are expressed in the reproductive system of human and mouse (Wichman et al., 2017), we postulated that *lncRNA8138.1* gene might play critical roles in reproduction and fertility.

Previous investigation indicated that lncRNA genetic variants play a central role in various biological processes (Wapinski & Chang, 2011), for instance SNP rs920778 in lncRNA HOTAIR, contributes to the risk of gastric cancer (Pan et al., 2016) and lncRNA SNP rs11655237 confers susceptibility to pancreatic cancer (Zheng et al., 2016). Mutations in lncRNA GAS8-AS1 were associated with papillary thyroid carcinoma (Chen et al., 2016). A study of whole-genome mutational landscape of liver cancer also discovered mutations in lncRNA NEAT1 and MALAT1 (Fujimoto et al., 2016). These studies have suggested an important role of nucleotide polymorphisms in lncRNAs.

Next, we explore the genetic variation of *lncRNA8138.1* gene in egg-laying hen populations. Generally, mutations are one of the forces of evolution because they fuel the variability in populations and thus enable evolutionary change (Loewe & Hill, 2010). Just like other genetic variations, SNPs can be directly detected by simple PCR amplification and agarose gel electrophoresis, making them convenient and practical. Therefore, SNP variants in the *lncRNA8138.1* gene were identified, and their associations with DF-trait investigated in a large commercial population of egg-laying hens.

Five novel mutations including r.4937159A>G, r.4937219T>C, r.4937258G>C, r.4937318C>G and g.4937319_4937325delinsTGTGTGG were identified in *lncRNA8138.1* gene, and a single PCR system was used to detect SNP r.4937159A>G. Three genotypes (AA, AG, and GG) were identified and statistical analysis indicates that SNP r.4937159A>G genotypes were not in HWE (*P* < 0.05) in laying hen populations, because of the lower number of observed AA and GG genotypes. Another one possible reason for this is rapid and effective selection, which could affect the allelic balance of the SNP locus (Crowley et al., 2015; Dunn et al., 2011). Differences between genotypes indicate that (GG vs AA) and (GG vs AG) genotypes were significantly different whereas no significant difference between AA vs AG genotypes for the DF-trait in the P1. The GG carriers had prolonged-DF in both P1 and P2 when compared to AA and AG genotypes (Table 2), suggesting that GG may be a favourable genotype for DF-trait in the egg-laying hens.

Advances in structural biology have made in the understanding of the molecular mechanism behind lncRNA function (Blythe, Fox & Bond, 2016; Novikova et al., 2013). To further understand *lncRNA8138.1* r.4937159A>G mutation structure in chicken; we predicted a common secondary structure between AA and GG carriers by RNA structure software version 6.1 and a 2-point mutation (AG_insertion) was found in GG carriers, and 2-point mutation (AG_deletion) in AA carriers were noted in the loop (Fig. 3). The 2-point mutations that exist in the AA and GG individuals can be used as a fingerprint to find the structure in thousands of other species.

Furthermore, the analysis of association between r.4937159A>G polymorphism and DF-trait investigated in a total of 1868 individuals of egg-laying hens shows significant correlation with DF-phenotypes; however, it can be considered that this locus is indeed correlated with the tested trait, especially in large population which improves the credibility of the test (*P* < 0.0001, *P* ≤ 0.05). The association analysis based on the large laying hen populations revealed r.4937159A>G polymorphism was strongly associated with DF-trait in hens, which was consistent with our previous investigations, suggesting that there are genetic variations that might impact *lncRNA8138.1* expression and function in egg-laying hens. Additionally, the fact that additive effect was significant for EN, FN and DN in egg-laying hens, indicating that *lncRNA8138.1* gene plays a vital role in regulating DF-trait.

## Conclusions

The biological role of *lncRNA8138.1* gene in regulating DF-trait also held while examining the presence of nucleotide variations. In the present study, four candidate SNPs and 7 nt indel were identified in the *lncRNA8138.1* gene; one of SNP (r.4937159A>G) was detected and found to be significantly associated with DF-trait (EN, FN and DN) in large egg-laying hen populations; indicating the emerging role of variants in long non-coding RNA might benefit the selection of birds for the marker-assisted breeding program. However, these findings extend our previous research on differential effects of *lncRNA8138.1* gene in egg-laying hens and invite future studies to investigate the *lncRNA8138.1* mutations in laying hen populations to enabling a better understanding of the genetic mechanism regulating DF-trait.

##  Supplemental Information

10.7717/peerj.7282/supp-1 File S1 Raw data of association at *lncRNA8138.1* gene

## Additional Information and Declarations

### Data Availability

The following information was supplied regarding data availability: Data is available at the NCBI database under accession number MK336169.
https://www.ncbi.nlm.nih.gov/nuccore/MK336169.
