# Supplementary material for: Association of single nucleotide polymorphism at long non-coding RNA 8138.1 with duration of fertility in egg-laying hens
Source: PeerJ. 2019 Jul 12;7:e7282. doi: 10.7717/peerj.7282 (PMC6628881; doi:10.7717/peerj.7282)
Supplement: File S1 [file peerj-07-7282-s001.docx]

**Association of Single Nucleotide Polymorphism at Long Non-Coding RNA 8138.1 with Duration of Fertility in Egg-laying Hens**

Adeyinka Abiola Adetula^1^, Syed Ali Azmal^1,2^ , Chenghao Sun^3^, Abdelmotaleb Ahmed Elokil^1,4^ and Shijun Li^1*^

^1^Key Laboratory of Agricultural Animal Genetics, Breeding, and Reproduction of the Ministry of Education, Huazhong Agricultural University, Wuhan, Hubei, China.

^2^Department of Livestock Services (DLS), Under the Ministry of Fisheries and Livestock (MOFL), Dhaka 1000, Bangladesh.

^3^Huadu Yukou Poultry Industry Co. Ltd, Beijing, China.

^4^Department of Animal Production, Faculty of Agriculture, Benha University, Moshtohor 13736, Egypt.

Corresponding Author: Shijun Li

No. 1, Shizishan Street, Hongshan District, Wuhan, 430070, China

Email address: [lishijun@mail.hzau.edu.cn](mailto:lishijun@mail.hzau.edu.cn)

**Supplementary File 1:** **Raw data of association at *lncRNA8138.1* gene.**

| **P1-CAGE** | **Wg_no** | **Avg. EN** | **Avg. FN** | **Avg. DN** | **(A/G) mutation** |
| --- | --- | --- | --- | --- | --- |
| 20000 | A1 | 15 | 10.333333 | 11 | GG |
| 20001 | A2 | 14 | 10 | 11.333333 | AG |
| 20002 | A3 | 15.333333 | 12.333333 | 12.333333 | AG |
| 20004 | A4 | 16.666667 | 9 | 11.333333 | AG |
| 20005 | A5 | 9 | 6.6666667 | 10.333333 | GG |
| 20007 | A6 | 7.6666667 | 2.3333333 | 7 | AG |
| 20100 | A7 | 11.666667 | 8.3333333 | 11 | AG |
| 20103 | A8 | 16 | 9.6666667 | 11 | AG |
| 20104 | A9 | 13.333333 | 8 | 11.333333 | GG |
| 20107 | A10 | 12.333333 | 9 | 10 | AA |
| 20108 | A12 | 11.333333 | 7 | 12 | AG |
| 20110 | A13 | 12.333333 | 8.3333333 | 10.333333 | AG |
| 20200 | A14 | 14.333333 | 12 | 12 | GG |
| 20201 | A15 | 4.3333333 | 4 | 5 | AG |
| 20202 | A16 | 3.6666667 | 3 | 5.6666667 | AG |
| 20204 | A17 | 15.666667 | 7.3333333 | 11.666667 | AG |
| 20205 | A18 | 14 | 10.333333 | 10.666667 | AG |
| 20208 | A19 | 14.666667 | 11.666667 | 11 | AG |
| 20210 | A20 | 14.666667 | 9.6666667 | 10 | AG |
| 20212 | A21 | 10.333333 | 8.3333333 | 10 | AG |
| 20214 | A23 | 11.333333 | 9.3333333 | 11.333333 | AA |
| 20216 | A24 | 11.666667 | 7 | 11.666667 | AG |
| 20300 | A25 | 15 | 9 | 11.333333 | AG |
| 20301 | A26 | 14.666667 | 8 | 10 | AG |
| 20302 | A27 | 13.666667 | 8.3333333 | 12 | AG |
| 20303 | A28 | 10 | 7.6666667 | 9 | GG |
| 20304 | A29 | 11.666667 | 6.6666667 | 10.333333 | AG |
| 20305 | A30 | 14.333333 | 9.6666667 | 11.666667 | AG |
| 20306 | A31 | 14.666667 | 10 | 12 | AG |
| 20307 | A32 | 15.666667 | 8 | 10.666667 | AG |
| 20308 | A33 | 14 | 11 | 11.666667 | AG |
| 20309 | A34 | 8.6666667 | 6.6666667 | 10 | AG |
| 20311 | A35 | 16 | 12.333333 | 11.333333 | AG |
| 20400 | A36 | 14 | 11 | 11.666667 | AG |
| 20403 | A37 | 14 | 10.666667 | 11.333333 | AA |
| 20404 | A38 | 15.666667 | 11 | 12 | AG |
| 20405 | A39 | 14.333333 | 7.6666667 | 10.333333 | AG |
| 20408 | A40 | 7.3333333 | 5.3333333 | 6.6666667 | AG |
| 20500 | A41 | 16 | 10 | 11 | AG |
| 20501 | A42 | 15 | 9 | 9.6666667 | AG |
| 20502 | A43 | 14 | 9.3333333 | 11 | AG |
| 20503 | A44 | 15.666667 | 13.333333 | 12.333333 | GG |
| 20504 | A45 | 12.333333 | 7 | 11.333333 | GG |
| 20506 | A46 | 11.333333 | 8.3333333 | 11 | AG |
| 20600 | A47 | 14.666667 | 9 | 10.333333 | AG |
| 20601 | A48 | 15.666667 | 11.666667 | 10 | AG |
| 20603 | B1 | 15.666667 | 11 | 11.333333 | GG |
| 20604 | B2 | 17 | 10.333333 | 11 | AG |
| 20605 | B3 | 16 | 10.666667 | 10.666667 | AA |
| 20606 | B4 | 13 | 8 | 11.666667 | AG |
| 20608 | B5 | 14 | 8.3333333 | 11.333333 | AG |
| 20609 | B6 | 13 | 9.3333333 | 11 | AA |
| 20610 | B7 | 14.333333 | 10 | 11.333333 | AG |
| 20611 | B8 | 14.333333 | 8.3333333 | 12 | AG |
| 20612 | B9 | 13.666667 | 8.6666667 | 11 | AA |
| 20613 | B10 | 15.666667 | 12.333333 | 11.333333 | GG |
| 20614 | B11 | 12.666667 | 8.6666667 | 11 | AG |
| 20700 | B12 | 15 | 10.333333 | 11.333333 | GG |
| 20702 | B13 | 15.666667 | 9.3333333 | 11.666667 | AG |
| 20703 | B14 | 9.3333333 | 4.3333333 | 9.6666667 | AG |
| 20706 | B15 | 13 | 11.333333 | 10 | AG |
| 20707 | B16 | 15.333333 | 11.666667 | 12 | AG |
| 20708 | B17 | 14 | 11 | 11 | AG |
| 20710 | B18 | 13.333333 | 8 | 10 | AG |
| 20712 | B19 | 14.333333 | 10 | 11.666667 | AG |
| 20802 | B20 | 16 | 10.666667 | 11.666667 | AG |
| 20803 | B21 | 0.3333333 | 0 | 0 | AG |
| 20804 | B22 | 16.333333 | 11.666667 | 11.666667 | AA |
| 20806 | B23 | 12.666667 | 8 | 11.666667 | AG |
| 20808 | B24 | 14.666667 | 11 | 11.666667 | AG |
| 20810 | B25 | 15.333333 | 12.333333 | 11 | AG |
| 20900 | B26 | 11 | 7.6666667 | 10 | GG |
| 20901 | B27 | 14.666667 | 7.6666667 | 11.333333 | AG |
| 20902 | B28 | 14 | 8 | 11.666667 | AA |
| 20904 | B29 | 14.333333 | 10.666667 | 11.666667 | AG |
| 20906 | B31 | 14.333333 | 11.333333 | 11 | AG |
| 21000 | B32 | 6.3333333 | 3.6666667 | 9 | AG |
| 21001 | B33 | 15 | 10.666667 | 11.333333 | AA |
| 21002 | B34 | 14 | 7.6666667 | 10.666667 | AG |
| 21003 | B35 | 7 | 3.3333333 | 6 | GG |
| 21005 | B36 | 11.666667 | 6 | 9.3333333 | GG |
| 21006 | B37 | 15.666667 | 9.6666667 | 11.333333 | AG |
| 21007 | B38 | 16.333333 | 8.6666667 | 8 | GG |
| 21008 | B39 | 11.333333 | 8 | 11.666667 | AG |
| 21011 | B40 | 15.666667 | 9.3333333 | 11 | AG |
| 21100 | B41 | 15 | 12 | 11 | AG |
| 21101 | B42 | 15.333333 | 8.6666667 | 9.3333333 | AG |
| 21102 | B43 | 14.333333 | 9.3333333 | 11.666667 | AG |
| 21104 | B44 | 14 | 10.333333 | 11.333333 | AG |
| 21105 | B45 | 14.333333 | 8.6666667 | 9.6666667 | AA |
| 21106 | B46 | 15.666667 | 7.6666667 | 9.6666667 | GG |
| 21107 | B47 | 13.333333 | 6 | 10.333333 | GG |
| 21108 | B48 | 16 | 12 | 12 | AG |
| 21110 | C1 | 16 | 10.333333 | 11.666667 | AG |
| 21112 | C2 | 14.666667 | 11 | 12 | AG |
| 21201 | C3 | 12.666667 | 10.333333 | 12 | AG |
| 21202 | C4 | 13.333333 | 9 | 10.666667 | AG |
| 21203 | C5 | 15.333333 | 9 | 10.333333 | AG |
| 21204 | C6 | 1.3333333 | 1.3333333 | 3 | AA |
| 21205 | C7 | 4.3333333 | 2.6666667 | 6.3333333 | AG |
| 21207 | C8 | 14.666667 | 12.666667 | 10.666667 | AG |
| 21208 | C9 | 15 | 8 | 11.333333 | AG |
| 21300 | C10 | 14.333333 | 11.333333 | 11.666667 | AG |
| 21301 | C11 | 0 | 0 | 0 | AA |
| 21302 | C12 | 12.666667 | 6.6666667 | 11 | AG |
| 21303 | C13 | 14 | 7.6666667 | 10 | AG |
| 21304 | C14 | 11.666667 | 10.333333 | 10 | AG |
| 21306 | C15 | 16.666667 | 12.666667 | 12.333333 | GG |
| 21401 | C16 | 13.333333 | 10 | 11.333333 | AG |
| 21402 | C17 | 14 | 10.666667 | 11.333333 | AG |
| 21403 | C18 | 15 | 7 | 11.333333 | AG |
| 21404 | C19 | 11.666667 | 8 | 11.666667 | AA |
| 21406 | C20 | 13.333333 | 11.333333 | 11.333333 | AG |
| 21407 | C21 | 15.666667 | 10 | 11.333333 | AG |
| 21409 | C22 | 15.333333 | 10.333333 | 11.333333 | GG |
| 21411 | C23 | 14.666667 | 11.333333 | 12.333333 | GG |
| 21500 | C24 | 14.333333 | 12 | 11 | GG |
| 21501 | C25 | 14.666667 | 9.6666667 | 11.666667 | AG |
| 21502 | C26 | 16 | 12.666667 | 11.333333 | AG |
| 21503 | C27 | 16.666667 | 10 | 11.666667 | AG |
| 21504 | C28 | 15.666667 | 8.3333333 | 11 | AG |
| 21505 | C29 | 14.666667 | 10.333333 | 11 | AG |
| 21507 | C30 | 15 | 10.666667 | 11.333333 | AG |
| 21509 | C31 | 14.666667 | 9.3333333 | 10.666667 | AG |
| 21511 | C32 | 11.333333 | 9 | 11.666667 | AG |
| 21515 | C33 | 5.6666667 | 3.6666667 | 8.6666667 | AA |
| 21600 | C34 | 13.666667 | 9.3333333 | 10 | AG |
| 21601 | C35 | 15.333333 | 10 | 10.333333 | AG |
| 21602 | C36 | 16.333333 | 10.333333 | 11.333333 | GG |
| 21700 | C37 | 11.666667 | 10.333333 | 10.333333 | AG |
| 21701 | C38 | 13.666667 | 7 | 12 | AG |
| 21702 | C39 | 15.666667 | 10.333333 | 11.666667 | AG |
| 21703 | C40 | 12 | 9.6666667 | 12.333333 | AA |
| 21706 | C41 | 15.666667 | 12.333333 | 11.666667 | GG |
| 21708 | C42 | 16.333333 | 12 | 12 | AA |
| 21709 | C43 | 0.3333333 | 0.3333333 | 4.3333333 | GG |
| 21800 | C44 | 16 | 9.3333333 | 11.333333 | AG |
| 21801 | C45 | 15 | 7 | 11.333333 | AG |
| 21802 | C46 | 14.666667 | 13.333333 | 12 | AG |
| 21803 | C47 | 15 | 11.666667 | 11.666667 | AG |
| 21804 | C48 | 15 | 11.666667 | 12 | AG |
| 21805 | D1 | 10 | 5.6666667 | 10.333333 | GG |
| 21809 | D2 | 12.666667 | 8.3333333 | 11 | AG |
| 21813 | D3 | 14.333333 | 9 | 12 | AG |
| 21900 | D4 | 14.333333 | 8.3333333 | 12.333333 | GG |
| 21902 | D5 | 4.6666667 | 2 | 8.3333333 | AG |
| 21904 | D6 | 15 | 10 | 12.333333 | AG |
| 21905 | D7 | 13.333333 | 11 | 10.333333 | AG |
| 21910 | D8 | 14.333333 | 8.6666667 | 11 | AG |
| 21911 | D9 | 15 | 10 | 11 | AG |
| 21912 | D10 | 15.333333 | 12.333333 | 11.666667 | GG |
| 22001 | D11 | 14.666667 | 6.6666667 | 10.333333 | AG |
| 22002 | D12 | 13 | 8 | 11.666667 | AG |
| 22003 | D13 | 16.333333 | 9.3333333 | 11 | AG |
| 22006 | D14 | 15 | 5.3333333 | 11.666667 | GG |
| 22010 | D15 | 15.333333 | 8.6666667 | 11 | AG |
| 22012 | D16 | 11.666667 | 5 | 9 | AA |
| 22100 | D17 | 15.333333 | 9 | 12.333333 | AG |
| 22101 | D18 | 13.333333 | 7.6666667 | 11.666667 | AG |
| 22102 | D19 | 15.333333 | 9.3333333 | 10.666667 | AG |
| 22103 | D20 | 11.666667 | 9.6666667 | 11 | GG |
| 22104 | D21 | 12.333333 | 8.3333333 | 11.666667 | GG |
| 22105 | D22 | 14.666667 | 8.6666667 | 11.333333 | AG |
| 22106 | D23 | 14.333333 | 10.333333 | 11.666667 | AG |
| 22107 | D24 | 15.666667 | 11.333333 | 12.333333 | AG |
| 22108 | D25 | 15 | 10.666667 | 12 | AG |
| 22110 | D26 | 14 | 10.666667 | 12 | AG |
| 22112 | D27 | 13.666667 | 9.6666667 | 11.666667 | AG |
| 22200 | D28 | 14 | 8.3333333 | 11 | AG |
| 22201 | D29 | 13 | 8 | 11.333333 | AG |
| 22202 | D30 | 14 | 9.3333333 | 11.333333 | AG |
| 22204 | D31 | 15 | 8.3333333 | 10.666667 | AG |
| 22205 | D32 | 14.666667 | 9.3333333 | 10.333333 | AG |
| 22206 | D33 | 14.666667 | 8 | 12 | AG |
| 22207 | D34 | 11.333333 | 8.6666667 | 11 | GG |
| 22210 | D35 | 15.333333 | 5.3333333 | 12 | AG |
| 22212 | D36 | 14.333333 | 11 | 11.333333 | AG |
| 22214 | D37 | 15.333333 | 10.666667 | 11.666667 | GG |
| 22300 | D38 | 13.666667 | 7.3333333 | 10.333333 | AG |
| 22301 | D39 | 16.333333 | 12.333333 | 12 | AG |
| 22303 | D40 | 13.333333 | 8.3333333 | 11.333333 | AG |
| 22304 | D41 | 13.666667 | 8.3333333 | 9.6666667 | AA |
| 22305 | D42 | 5.3333333 | 3 | 7 | AG |
| 22307 | D43 | 15 | 9.6666667 | 10 | AG |
| 22400 | D44 | 15.666667 | 12 | 11.666667 | AG |
| 22401 | D45 | 15.333333 | 8.3333333 | 11.333333 | AG |
| 22402 | D46 | 12.666667 | 8.3333333 | 10.666667 | AG |
| 22403 | D47 | 15.333333 | 9.6666667 | 11.666667 | AA |
| 22404 | D48 | 16.333333 | 10 | 10.666667 | AG |
| 22405 | E1 | 15.666667 | 12.333333 | 12 | AG |
| 22408 | E2 | 13.666667 | 5.6666667 | 11 | AA |
| 22410 | E3 | 8 | 6 | 12 | GG |
| 22501 | E5 | 13.666667 | 9.3333333 | 11.333333 | AG |
| 22502 | E6 | 13.666667 | 9.6666667 | 10.666667 | AA |
| 22504 | E7 | 14.333333 | 11 | 11.333333 | AG |
| 22505 | E8 | 14 | 10 | 11 | AG |
| 22506 | E9 | 12.666667 | 8.3333333 | 11 | AG |
| 22507 | E10 | 13.666667 | 10.666667 | 10.333333 | AA |
| 22508 | E11 | 15.666667 | 10.333333 | 11 | AG |
| 22510 | E12 | 14.666667 | 8 | 10.666667 | AA |
| 22600 | E13 | 15.333333 | 12 | 12.333333 | AG |
| 22601 | E14 | 15.333333 | 11.333333 | 11.666667 | AG |
| 22602 | E15 | 14.666667 | 10 | 11.666667 | AG |
| 22604 | E16 | 14.666667 | 9 | 10.333333 | AG |
| 22606 | E17 | 14.333333 | 8.3333333 | 11.333333 | AG |
| 22608 | E18 | 14.333333 | 8 | 11.333333 | AG |
| 22610 | E19 | 11.666667 | 5.6666667 | 11.333333 | AG |
| 22612 | E20 | 15 | 11 | 10.333333 | AG |
| 22614 | E21 | 15 | 11.666667 | 10.333333 | AG |
| 22700 | E22 | 15 | 11 | 11 | AG |
| 22701 | E23 | 15.666667 | 12.666667 | 12.333333 | AG |
| 22704 | E24 | 11 | 8.3333333 | 10 | AA |
| 22705 | E25 | 14.666667 | 8.6666667 | 9.6666667 | GG |
| 22706 | E26 | 14.333333 | 9.6666667 | 11.333333 | GG |
| 22707 | E27 | 13.666667 | 8 | 12.333333 | AG |
| 22708 | E28 | 14.666667 | 8.3333333 | 11.666667 | GG |
| 22709 | E29 | 14.333333 | 10 | 12 | AG |
| 22710 | E30 | 10.666667 | 7 | 9.6666667 | GG |
| 22800 | E31 | 16 | 12.666667 | 11.333333 | AG |
| 22801 | E32 | 14.333333 | 10 | 10.666667 | AG |
| 22802 | E33 | 12 | 8.3333333 | 11.333333 | AG |
| 22803 | E34 | 13 | 7.3333333 | 11 | AG |
| 22805 | E35 | 4.3333333 | 3.3333333 | 7.6666667 | AG |
| 22806 | E36 | 13.333333 | 9 | 11 | AG |
| 22807 | E37 | 16.666667 | 12 | 11.666667 | AG |
| 22809 | E38 | 14.333333 | 10 | 10.333333 | AG |
| 22813 | E39 | 15.333333 | 10.333333 | 11.666667 | AG |
| 23200 | E40 | 9.6666667 | 6.3333333 | 8 | AG |
| 23201 | E41 | 15 | 11.333333 | 12.333333 | AG |
| 23202 | E42 | 5.6666667 | 3.6666667 | 6 | AG |
| 23203 | E43 | 17 | 12.333333 | 11 | AG |
| 23204 | E44 | 14.333333 | 11 | 11.333333 | AG |
| 23205 | E45 | 16 | 11.666667 | 11.333333 | AG |
| 23206 | E46 | 9 | 6.3333333 | 7.6666667 | AG |
| 23207 | E47 | 14.333333 | 9 | 11.333333 | AG |
| 23210 | E48 | 1.3333333 | 1 | 3.6666667 | AG |
| 23211 | F1 | 13.666667 | 6.6666667 | 9.6666667 | AG |
| 23214 | F2 | 15.666667 | 11.666667 | 12 | AG |
| 23300 | F3 | 14.666667 | 9 | 10.666667 | AG |
| 23302 | F4 | 16 | 10.666667 | 11 | AG |
| 23304 | F5 | 16 | 8 | 9 | AG |
| 23306 | F6 | 14 | 12.333333 | 12 | AG |
| 23307 | F7 | 14.333333 | 8.3333333 | 11 | AG |
| 23308 | F8 | 15.333333 | 12 | 12.333333 | AG |
| 23309 | F9 | 2.3333333 | 2 | 3 | GG |
| 23310 | F10 | 16 | 13 | 12.333333 | AG |
| 23400 | F11 | 15.333333 | 11.333333 | 11.333333 | GG |
| 23403 | F12 | 15 | 10.666667 | 8.6666667 | AG |
| 23404 | F13 | 13.666667 | 10.666667 | 11 | AG |
| 23405 | F14 | 15 | 10.666667 | 10.666667 | AG |
| 23407 | F15 | 13.333333 | 6.6666667 | 10.666667 | AG |
| 23500 | F16 | 14 | 11 | 12 | AG |
| 23501 | F17 | 13 | 6 | 10.333333 | AG |
| 23502 | F18 | 16.333333 | 11.666667 | 11 | AG |
| 23503 | F19 | 14 | 10.333333 | 11 | AG |
| 23504 | F20 | 4.6666667 | 3 | 5.6666667 | AA |
| 23505 | F21 | 14.333333 | 10.666667 | 12 | AA |
| 23506 | F22 | 14 | 9.3333333 | 11.666667 | AG |
| 23507 | F23 | 16 | 12.333333 | 11.333333 | AG |
| 23508 | F24 | 16.333333 | 13.666667 | 15 | AG |
| 23509 | F25 | 13.666667 | 10.333333 | 10.333333 | AG |
| 23510 | F26 | 16 | 7.3333333 | 10.333333 | AG |
| 23600 | F27 | 16.666667 | 8.6666667 | 11.666667 | AG |
| 23601 | F28 | 12.666667 | 6 | 11.333333 | AG |
| 23602 | F29 | 15.333333 | 11.666667 | 11.333333 | AG |
| 23603 | F30 | 14.666667 | 12.666667 | 12.333333 | AG |
| 23604 | F31 | 11.666667 | 6.6666667 | 9 | AA |
| 23607 | F32 | 14 | 10.333333 | 12 | AA |
| 23609 | F33 | 14.333333 | 5 | 11.666667 | AA |
| 23700 | F34 | 13.333333 | 10.666667 | 11.333333 | AG |
| 23701 | F35 | 13.666667 | 11 | 11.333333 | AG |
| 23702 | F36 | 15 | 12 | 12.333333 | GG |
| 23703 | F37 | 16.666667 | 7.3333333 | 11.333333 | AG |
| 23704 | F38 | 16.666667 | 10.666667 | 10.333333 | AG |
| 23705 | F39 | 14.666667 | 11.333333 | 11.666667 | AG |
| 23706 | F40 | 14.666667 | 7 | 10.666667 | GG |
| 23708 | F41 | 14.333333 | 9.6666667 | 11 | AG |
| 23709 | F42 | 15 | 11.666667 | 11.333333 | AG |
| 23710 | F43 | 14.333333 | 9.3333333 | 11 | AA |
| 23711 | F44 | 14.333333 | 8.3333333 | 9.6666667 | AA |
| 23712 | F45 | 15.666667 | 12.333333 | 11.333333 | GG |
| 23713 | F46 | 14 | 12 | 11.333333 | AG |
| 23714 | F47 | 11.666667 | 7 | 11.666667 | AA |
| 23715 | F48 | 16 | 9.6666667 | 12 | AG |
| 23716 | G1 | 12.666667 | 7.6666667 | 10.666667 | AG |
| 23717 | G2 | 14.666667 | 9.6666667 | 11 | AG |
| 23718 | G3 | 16.333333 | 11 | 11.666667 | AG |
| 23719 | G4 | 10.666667 | 9.3333333 | 10.666667 | AG |
| 23720 | G5 | 15.333333 | 10.666667 | 10.333333 | AA |
| 23721 | G6 | 15 | 5 | 9 | GG |
| 23722 | G7 | 16.333333 | 11.333333 | 10.666667 | GG |
| 23723 | G8 | 16 | 12.333333 | 10.666667 | AA |
| 23724 | G9 | 9.3333333 | 8.3333333 | 9 | AG |
| 23725 | G10 | 13.333333 | 7.3333333 | 11.333333 | GG |
| 23726 | G11 | 16 | 12.666667 | 11.666667 | AG |
| 23727 | G12 | 15.333333 | 7.6666667 | 9.6666667 | AG |
| 23728 | G13 | 15.333333 | 11.333333 | 12 | AA |
| 23729 | G14 | 15.666667 | 11.333333 | 11.333333 | AG |
| 23730 | G15 | 14 | 9.6666667 | 10.666667 | AA |
| 23731 | G16 | 17 | 10 | 11 | AG |
| 23732 | G17 | 15.666667 | 11 | 10.333333 | AG |
| 23733 | G18 | 15.666667 | 10.333333 | 11.333333 | AG |
| 23734 | G19 | 16.333333 | 9.6666667 | 11.333333 | AG |
| 23735 | G20 | 13.333333 | 10.333333 | 11.333333 | AG |
| 23736 | G21 | 11.666667 | 7.3333333 | 10.666667 | AG |
| 23737 | G22 | 15.333333 | 11 | 12.333333 | AG |
| 23738 | G23 | 14.333333 | 11.333333 | 11.333333 | AG |
| 23739 | G24 | 15.333333 | 10.333333 | 10.666667 | AG |
| 23740 | G25 | 14.666667 | 10.666667 | 11.666667 | GG |
| 23741 | G26 | 15.666667 | 10.333333 | 10.666667 | AA |
| 23742 | G27 | 6.6666667 | 3.6666667 | 8 | GG |
| 23743 | G28 | 15 | 10.666667 | 11.666667 | AG |
| 23744 | G30 | 15.666667 | 9 | 9 | GG |
| 23745 | G31 | 15.666667 | 9.6666667 | 11 | AG |
| 23746 | G32 | 15.333333 | 10.333333 | 9.6666667 | AA |
| 23747 | G33 | 14.333333 | 9.6666667 | 11.666667 | AG |
| 23748 | G34 | 2 | 1.3333333 | 4 | GG |
| 23749 | G35 | 15.666667 | 13 | 11.666667 | AG |
|  |  |  |  |  |  |
| 23750 | G36 | 14.666667 | 11 | 12 | AG |
| 23751 | G37 | 16 | 9.3333333 | 11.333333 | GG |
| 23752 | G38 | 16.666667 | 12.333333 | 11.666667 | AG |
| 23753 | G39 | 13.666667 | 8.6666667 | 12 | AG |
| 23754 | G40 | 15.333333 | 8.3333333 | 10.333333 | AA |
| 23755 | G41 | 16 | 8.6666667 | 10.666667 | GG |
| 23756 | G42 | 14.333333 | 9 | 11 | AA |
| 23757 | G43 | 14 | 8.6666667 | 12 | AG |
| 23758 | G44 | 17 | 8.6666667 | 10.666667 | AG |
| 23759 | G45 | 14 | 10.666667 | 11 | AA |
| 23760 | G46 | 16 | 11.333333 | 11.333333 | AG |
| 23761 | G47 | 11.666667 | 7.3333333 | 10.666667 | AG |
| 23762 | G48 | 12.666667 | 8 | 11 | AG |
| 23763 | H1 | 14.666667 | 9.3333333 | 11.333333 | AG |
| 23764 | H2 | 16.666667 | 12 | 10.666667 | AG |
| 23765 | H3 | 13.333333 | 10.333333 | 11.333333 | AG |
| 23766 | H4 | 16.333333 | 10.666667 | 11 | AG |
| 23767 | H5 | 10 | 6.6666667 | 11.666667 | AG |
| 23768 | H6 | 15.333333 | 8.6666667 | 10 | AG |
| 23769 | H7 | 14.666667 | 8.6666667 | 11.333333 | AG |
| 23770 | H8 | 12.333333 | 10.333333 | 12 | AA |
| 23771 | H9 | 14 | 10.666667 | 10.333333 | AG |
| 23772 | H10 | 14.666667 | 9.3333333 | 11.666667 | AG |
| 23773 | H11 | 12.666667 | 5.6666667 | 10 | AG |
| 23774 | H12 | 13 | 8.6666667 | 11.666667 | GG |
| 23775 | H13 | 15 | 12 | 10.666667 | AG |
| 23776 | H14 | 7 | 5.6666667 | 7.3333333 | AG |
| 23777 | H15 | 15.666667 | 14 | 14 | AA |
| 23778 | H16 | 11.666667 | 8 | 11.333333 | AA |
| 23779 | H17 | 15.333333 | 10 | 11.666667 | AA |
| 23780 | H18 | 3 | 2 | 3.3333333 | AG |
| 23781 | H19 | 15.666667 | 11.666667 | 11.666667 | AG |
| 23782 | H20 | 14.333333 | 7.3333333 | 11.333333 | AG |
| 23783 | H21 | 13 | 7 | 11 | AG |
| 23784 | H22 | 14.333333 | 8.3333333 | 12.333333 | AG |
| 23785 | H23 | 13 | 7 | 10.666667 | AG |
| 23786 | H25 | 13 | 8.3333333 | 11 | AG |
| 23787 | H26 | 13.666667 | 5.6666667 | 9.6666667 | AG |
| 23788 | H27 | 11 | 6.3333333 | 10.333333 | AG |
| 23789 | H28 | 15 | 9.6666667 | 11 | AG |
| 23790 | H29 | 14.666667 | 9.6666667 | 11.666667 | AG |
| 23791 | H30 | 9.6666667 | 5.3333333 | 8 | AG |
| 23792 | H31 | 5 | 3 | 6 | AG |
| 23793 | H32 | 14.333333 | 10 | 11 | AA |
| 23794 | H33 | 3 | 1.6666667 | 5.6666667 | GG |
| 23795 | H34 | 11.333333 | 5.6666667 | 12 | AG |
| 23796 | H35 | 15.333333 | 12.333333 | 11.333333 | GG |
| 23797 | H36 | 15.333333 | 11.666667 | 11.333333 | AG |
| 23798 | H37 | 16 | 11.333333 | 11.333333 | AG |
| 23799 | H38 | 14.666667 | 10.333333 | 11.333333 | AG |
| 23800 | H39 | 16 | 7.3333333 | 11.333333 | AG |
| 23801 | H40 | 15 | 8 | 12 | AA |
| 23802 | H41 | 7.3333333 | 6.6666667 | 8.3333333 | AA |
| 23803 | H42 | 15 | 13.666667 | 16 | AG |
| 23804 | H43 | 15.333333 | 11.666667 | 10.666667 | AG |
| 23805 | H44 | 15.333333 | 11 | 11.333333 | AG |
| 23806 | H45 | 14.666667 | 7.3333333 | 11.666667 | AG |
| 23807 | H46 | 16 | 12.333333 | 12 | GG |
| 23808 | H47 | 15.333333 | 9.6666667 | 12 | AG |
| 23809 | H48 | 10.666667 | 8.6666667 | 11.333333 | AG |
| 23810 | I1 | 14 | 12.333333 | 12.333333 | AG |
| 23811 | I2 | 15.666667 | 13.666667 | 15 | AA |
| 23812 | I3 | 10 | 7.6666667 | 9.3333333 | GG |
| 23813 | I4 | 15.333333 | 10.666667 | 11.666667 | AG |
| 23814 | I5 | 15 | 8.6666667 | 12 | AA |
| 23815 | I6 | 16 | 13.333333 | 11.666667 | AG |
| 23816 | I7 | 14.666667 | 7.6666667 | 10.666667 | AG |
| 23817 | I8 | 15.333333 | 12 | 12 | GG |
| 23818 | I9 | 13.666667 | 8.6666667 | 11.333333 | AG |
| 23819 | I10 | 13.666667 | 10 | 11.333333 | AA |
| 23820 | I11 | 15.666667 | 9.6666667 | 10.333333 | AA |
| 23821 | I12 | 9.3333333 | 7.6666667 | 7.6666667 | AG |
| 23822 | I13 | 15 | 8.3333333 | 10.666667 | GG |
| 23823 | I14 | 16.333333 | 9.6666667 | 10.666667 | GG |
| 23824 | I15 | 15.666667 | 13.666667 | 14.3333 | GG |
| 23825 | I16 | 15.666667 | 11.333333 | 11.666667 | GG |
| 23826 | I17 | 12.666667 | 9 | 11 | AG |
| 23827 | I18 | 15.666667 | 12.333333 | 11 | GG |
| 23828 | I19 | 15 | 8.6666667 | 11 | GG |
| 23829 | I20 | 12.666667 | 9 | 9 | AG |
| 23830 | I21 | 16 | 10 | 11.333333 | GG |
| 23831 | I22 | 14 | 11 | 11 | AG |
| 23832 | I23 | 13.333333 | 8.3333333 | 10 | AA |
| 23833 | I24 | 15.666667 | 9.6666667 | 10.666667 | AG |
| 23834 | I25 | 12.333333 | 8 | 11.666667 | AG |
| 23835 | I26 | 14 | 10.666667 | 11.666667 | AG |
| 23836 | I27 | 14.333333 | 9 | 11 | AG |
| 23837 | I28 | 14.666667 | 12.666667 | 11.333333 | AG |
| 23838 | I29 | 16 | 10.666667 | 11.666667 | GG |
| 23839 | I30 | 17 | 11 | 10.666667 | GG |
| 23840 | I31 | 14.666667 | 11.333333 | 11.666667 | AG |
| 23841 | I32 | 15.666667 | 9.3333333 | 11.666667 | GG |
| 23842 | I33 | 16.333333 | 8.3333333 | 12 | GG |
| 23843 | I34 | 15.333333 | 10 | 11 | GG |
| 23844 | I35 | 14.333333 | 7.3333333 | 10.333333 | AG |
| 23845 | I36 | 13 | 7.6666667 | 12.333333 | AG |
| 23846 | I37 | 13.333333 | 10 | 12 | AG |
| 23847 | I38 | 16.333333 | 11.666667 | 11.666667 | GG |
| 23848 | I39 | 14.666667 | 7.6666667 | 10.333333 | AG |
| 23849 | I40 | 16.666667 | 11.333333 | 10.666667 | GG |
| 23850 | I41 | 11.666667 | 9.6666667 | 12 | AG |
| 23851 | I42 | 16 | 10.666667 | 10.666667 | GG |
| 23852 | I43 | 15.333333 | 13 | 11.333333 | GG |
| 23853 | I44 | 14.666667 | 8.6666667 | 10 | AG |
| 23854 | I45 | 14.333333 | 11.666667 | 11.333333 | AA |
| 23855 | I46 | 14 | 11 | 12 | AG |
| 23856 | I47 | 14.666667 | 9.6666667 | 10.333333 | AG |
| 23857 | I48 | 0 | 0 | 0 | AG |
| 23858 | J1 | 15.666667 | 9.6666667 | 11.333333 | GG |
| 23859 | J2 | 16.333333 | 13 | 12 | GG |
| 23860 | J3 | 12.666667 | 7.6666667 | 10.666667 | AA |
| 23861 | J4 | 3.6666667 | 1.3333333 | 6.6666667 | AA |
| 23862 | J5 | 15 | 7.6666667 | 10.666667 | AA |
| 23863 | J6 | 12 | 6.3333333 | 9.6666667 | AG |
| 23864 | J7 | 14 | 6.3333333 | 10.666667 | AG |
| 23865 | J8 | 16.666667 | 8.6666667 | 11 | GG |
| 23866 | J9 | 11.666667 | 9 | 11 | AG |
| 23867 | J10 | 14.333333 | 7.6666667 | 11.333333 | AG |
| 23868 | J11 | 11.666667 | 7.6666667 | 11.333333 | AG |
| 23869 | J12 | 15.666667 | 9 | 11.666667 | GG |
| 23870 | J13 | 13.666667 | 10 | 11.333333 | AG |
| 23871 | J14 | 15 | 10.666667 | 12.333333 | GG |
| 23872 | J15 | 15.333333 | 9 | 11.666667 | GG |
| 23873 | J16 | 15 | 8.3333333 | 11 | AA |
| 23874 | J17 | 11.666667 | 9 | 10.333333 | AG |
| 23875 | J18 | 15.333333 | 11.666667 | 12 | AG |
| 23876 | J19 | 14.333333 | 9.6666667 | 11.333333 | AA |
| 23877 | J20 | 13 | 12.333333 | 11.333333 | AG |
| 23878 | J21 | 13 | 11 | 11.666667 | AG |
| 23879 | J22 | 15 | 13 | 11.666667 | AG |
| 23880 | J23 | 14.333333 | 11 | 11 | AG |
| 23881 | J24 | 13 | 10.333333 | 12.333333 | AG |
| 23882 | J25 | 15 | 11.666667 | 11 | AA |
| 23883 | J26 | 14 | 7 | 10 | AG |
| 23884 | J27 | 15 | 11 | 11 | GG |
| 23885 | J28 | 3 | 2.6666667 | 3.3333333 | AG |
| 23886 | J29 | 14.666667 | 12.333333 | 12.333333 | AG |
| 23887 | J30 | 16.333333 | 10.333333 | 11.333333 | GG |
| 23888 | J31 | 8.3333333 | 2.6666667 | 9.3333333 | AA |
| 23889 | J32 | 16 | 11.666667 | 11.333333 | GG |
| 23890 | J33 | 11.333333 | 9.3333333 | 11.333333 | AA |
| 23891 | J34 | 7.3333333 | 4.3333333 | 7.3333333 | AG |
| 23892 | J35 | 14 | 11 | 11.333333 | AG |
| 23893 | J36 | 15.666667 | 13 | 11.333333 | GG |
| 23894 | J37 | 13.666667 | 8.3333333 | 11.666667 | AG |
| 23895 | J38 | 11.333333 | 8.6666667 | 10.666667 | AG |
| 23896 | J39 | 12.666667 | 9 | 10.666667 | AA |
| 23897 | J40 | 11.666667 | 7.6666667 | 11 | AA |
| 23898 | J41 | 16 | 9.6666667 | 11.666667 | GG |
| 23899 | J42 | 14.666667 | 9.6666667 | 11.333333 | AG |
| 23900 | J43 | 15.333333 | 9.6666667 | 11.333333 | GG |
| 23901 | J44 | 15.333333 | 10.666667 | 10.666667 | GG |
| 23902 | J45 | 13.333333 | 9 | 11 | GG |
| 23903 | J46 | 14.333333 | 10.666667 | 11.666667 | AG |
| 23904 | J47 | 16.333333 | 10.666667 | 11 | GG |
| 23905 | J48 | 14 | 10 | 11 | AG |
| 23906 | K1 | 13.666667 | 10.666667 | 11.666667 | AG |
| 23907 | K2 | 15.666667 | 11 | 11.333333 | GG |
| 23908 | K3 | 16.666667 | 12.666667 | 11.666667 | GG |
| 23909 | K4 | 16 | 10 | 12 | GG |
| 23910 | K5 | 12.333333 | 8 | 11.333333 | AG |
| 23911 | K6 | 11.666667 | 7.3333333 | 11.333333 | AG |
| 23912 | K7 | 14.666667 | 7.3333333 | 10.666667 | AG |
| 23913 | K8 | 14 | 9 | 11.333333 | AG |
| 23914 | K9 | 12 | 9 | 11.333333 | AG |
| 23915 | K10 | 16 | 9.3333333 | 10.333333 | GG |
| 23916 | K11 | 13.666667 | 7 | 11 | AA |
| 23917 | K12 | 13 | 7.6666667 | 11.333333 | AG |
| 23918 | K13 | 13.666667 | 10 | 11.333333 | AG |
| 23919 | K14 | 14.333333 | 10.666667 | 11.666667 | AG |
| 23920 | K15 | 15.333333 | 13.333333 | 12.333333 | GG |
| 23921 | K16 | 8 | 6.6666667 | 8.3333333 | AG |
| 23922 | K17 | 14 | 10.666667 | 11.666667 | AG |
| 23923 | K18 | 14 | 10 | 10.333333 | AG |
| 23924 | K19 | 15.333333 | 8.6666667 | 11.333333 | GG |
| 23925 | K20 | 12.666667 | 9 | 11.666667 | AG |
| 23926 | K21 | 12.333333 | 7.3333333 | 10.333333 | AG |
| 23927 | K22 | 16 | 11.666667 | 11.666667 | GG |
| 23928 | K23 | 14.333333 | 8 | 11.666667 | AA |
| 23929 | K24 | 14.666667 | 9.3333333 | 10.333333 | AG |
| 23930 | K25 | 16.666667 | 11 | 12 | GG |
| 23931 | K26 | 16 | 9.6666667 | 11.666667 | GG |
| 23932 | K27 | 15 | 9.3333333 | 11.333333 | AG |
| 23933 | K28 | 15.666667 | 10 | 11.333333 | GG |
| 23934 | K29 | 14.333333 | 10.666667 | 11.666667 | AG |
| 23935 | K30 | 13.333333 | 5.3333333 | 11.666667 | AG |
| 23936 | K31 | 15.666667 | 10 | 11.333333 | GG |
| 23937 | K32 | 1 | 0.3333333 | 0 | AG |
| 23938 | K34 | 12 | 9 | 11.333333 | AG |
| 23939 | K35 | 15 | 10.333333 | 10.333333 | GG |
| 23940 | K36 | 12.666667 | 7 | 11.333333 | AG |
| 23941 | K37 | 12.666667 | 7 | 9.6666667 | AG |
| 23942 | K38 | 14 | 10.666667 | 11 | AA |
| 23943 | K39 | 15.333333 | 7.6666667 | 11 | GG |
| 23944 | K40 | 15 | 10.666667 | 10.666667 | GG |
| 23945 | K41 | 14.666667 | 7 | 11.666667 | AG |
| 23946 | K42 | 15 | 12.333333 | 11.666667 | AG |
| 23947 | K43 | 15 | 10.333333 | 10.666667 | GG |
| 23948 | K44 | 16.333333 | 7.3333333 | 12.333333 | GG |
| 23949 | K45 | 16 | 11.333333 | 11.666667 | GG |
| 23950 | K46 | 15.666667 | 13.333333 | 12.333333 | GG |
| 23951 | K47 | 0 | 0 | 0 | AG |
| 23952 | K48 | 12 | 8 | 9.3333333 | AG |
| 23953 | L1 | 3.6666667 | 2.3333333 | 3.3333333 | AA |
| 23954 | L2 | 15.333333 | 10.666667 | 12.333333 | GG |
| 23955 | L4 | 14.333333 | 10.333333 | 10.666667 | AG |
| 23956 | L5 | 16 | 10 | 12.333333 | GG |
| 23957 | L6 | 16.333333 | 10 | 11 | GG |
| 23958 | L7 | 14.333333 | 10.333333 | 11.333333 | AA |
| 23959 | L8 | 14.333333 | 6.6666667 | 10.333333 | AA |
| 23960 | L9 | 16 | 10 | 12.333333 | GG |
| 23961 | L10 | 15.666667 | 8 | 11.333333 | GG |
| 23962 | L11 | 12 | 8.6666667 | 11.666667 | AG |
| 23963 | L12 | 15.333333 | 3.3333333 | 9 | AG |
| 23964 | L13 | 13.333333 | 10 | 10.333333 | AG |
| 23965 | L14 | 15 | 9 | 11.666667 | GG |
| 23966 | L15 | 15 | 10 | 12 | AG |
| 23967 | L16 | 14.333333 | 9.3333333 | 10.333333 | AG |
| 23968 | L17 | 10.666667 | 6 | 7.3333333 | AG |
| 23969 | L18 | 16 | 10.333333 | 11.666667 | GG |
| 23970 | L19 | 15.666667 | 7.6666667 | 9.3333333 | GG |
| 23971 | L20 | 14 | 7.3333333 | 12 | AG |
| 23972 | L21 | 14.666667 | 8 | 11.666667 | AG |
| 23973 | L22 | 14.666667 | 8.3333333 | 11.333333 | AG |
| 23974 | L23 | 12.333333 | 6 | 9.3333333 | AG |
| 23975 | L24 | 15.333333 | 10.666667 | 11.666667 | AG |
| 23976 | L25 | 11.666667 | 6.3333333 | 10.333333 | AG |
| 23977 | L26 | 1.3333333 | 1 | 8.6666667 | AG |
| 23978 | L27 | 15.666667 | 8.6666667 | 10 | GG |
| 23979 | L28 | 0.3333333 | 0 | 0 | AG |
| 23980 | L29 | 12 | 8 | 10.333333 | AG |
| 23981 | L30 | 17 | 9 | 11.333333 | GG |
| 23982 | L31 | 10.333333 | 4.6666667 | 12 | AG |
| 23983 | L32 | 14.666667 | 10.666667 | 11.666667 | AG |
| 23984 | L33 | 14.666667 | 7.6666667 | 11 | AG |
| 23985 | L34 | 14 | 11 | 11.333333 | AG |
| 23986 | L35 | 11.333333 | 8.6666667 | 10.666667 | AG |
| 23987 | L36 | 12 | 7.3333333 | 9.6666667 | AG |
| 23988 | L37 | 14 | 9.6666667 | 10.333333 | AG |
| 23989 | L38 | 13.666667 | 10 | 11 | AG |
| 23990 | L39 | 11.666667 | 4 | 9.6666667 | AG |
| 23991 | L40 | 13.333333 | 10 | 11.333333 | AG |
| 23992 | L41 | 13.333333 | 7.3333333 | 11 | AG |
| 23993 | L42 | 16 | 5.6666667 | 11.333333 | GG |
| 23994 | L43 | 2.3333333 | 1.3333333 | 8 | AG |
| 23995 | L44 | 13.333333 | 8.6666667 | 11.666667 | AG |
| 23996 | L45 | 17 | 14 | 15.3333 | GG |
| 23997 | L46 | 14.666667 | 8 | 11.333333 | AG |
| 23998 | L47 | 14.666667 | 8 | 11 | GG |
| 23999 | L48 | 14 | 8.6666667 | 11.666667 | AG |
| 24000 | M1 | 13.666667 | 4.3333333 | 10 | AG |
| 24001 | M2 | 16.333333 | 9.6666667 | 12.333333 | GG |
| 24002 | M3 | 15.666667 | 10.666667 | 11.666667 | GG |
| 24003 | M4 | 13.333333 | 7.6666667 | 11.333333 | AG |
| 24004 | M5 | 14.666667 | 11.666667 | 12 | GG |
| 24005 | M6 | 13.333333 | 7.6666667 | 9.6666667 | AG |
| 24006 | M7 | 14 | 10.666667 | 11.666667 | AG |
| 24007 | M8 | 15.333333 | 11 | 11.666667 | GG |
| 24008 | M9 | 8.3333333 | 6 | 7.3333333 | AG |
| 24009 | M10 | 14.666667 | 7.3333333 | 11.333333 | AG |
| 24010 | M11 | 16.666667 | 8.6666667 | 11.666667 | GG |
| 24011 | M12 | 14 | 8.6666667 | 10.666667 | AG |
| 24012 | M13 | 13.333333 | 9 | 11.333333 | AG |
| 24013 | M14 | 1.6666667 | 0.6666667 | 0.6666667 | AG |
| 24014 | M15 | 11.666667 | 9.6666667 | 12 | AG |
| 24015 | M16 | 13 | 9.3333333 | 11.333333 | AG |
| 24016 | M17 | 15.666667 | 9.6666667 | 10.333333 | GG |
| 24017 | M18 | 13.333333 | 6.6666667 | 11 | AG |
| 24018 | M19 | 14.666667 | 7.6666667 | 10.333333 | AG |
| 24019 | M20 | 15.666667 | 8.6666667 | 11 | GG |
| 24020 | M21 | 15 | 7 | 11.333333 | AG |
| 24021 | M22 | 14 | 9 | 12.333333 | AG |
| 24022 | M23 | 13.666667 | 10.333333 | 11.333333 | AG |
| 24023 | M24 | 14.333333 | 8.6666667 | 12.333333 | GG |
| 24024 | M25 | 12.666667 | 8.6666667 | 11 | AG |
| 24025 | M26 | 16.333333 | 11.333333 | 10 | GG |
| 24026 | M27 | 14 | 8.3333333 | 10.666667 | AG |
| 24027 | M28 | 13.666667 | 11.333333 | 11.666667 | AG |
| 24028 | M29 | 14.333333 | 10 | 11.333333 | AG |
| 24029 | M30 | 16.666667 | 13.333333 | 11 | GG |
| 24030 | M31 | 12.666667 | 10.333333 | 12 | AG |
| 24031 | M32 | 1 | 1 | 8.3333333 | AG |
| 24032 | M33 | 15.666667 | 11.666667 | 11.666667 | GG |
| 24033 | M34 | 13.666667 | 8.3333333 | 11.666667 | AG |
| 24034 | M35 | 11 | 6 | 11.333333 | AG |
| 24035 | M36 | 17 | 6.3333333 | 10 | GG |
| 24036 | M37 | 2.6666667 | 0.3333333 | 1 | AG |
| 24037 | M38 | 15.333333 | 11.666667 | 12 | GG |
| 24038 | M39 | 15.666667 | 10 | 11 | GG |
| 24039 | M40 | 15.333333 | 11.333333 | 12.333333 | GG |
| 24040 | M41 | 2 | 2 | 3.3333333 | AG |
| 24041 | M42 | 16 | 6.6666667 | 9.6666667 | GG |
| 24042 | M43 | 15.333333 | 5.3333333 | 10.666667 | GG |
| 24043 | M44 | 16.666667 | 9 | 11.666667 | GG |
| 24044 | M45 | 9.3333333 | 7.3333333 | 11.333333 | AA |
| 24045 | M46 | 15 | 8.3333333 | 11.666667 | GG |
| 24046 | M47 | 15.333333 | 9.6666667 | 11.333333 | GG |
| 24047 | M48 | 8.6666667 | 6.6666667 | 7.6666667 | AG |
| 24048 | N1 | 7.6666667 | 2 | 5 | AG |
| 24049 | N2 | 13.666667 | 9.6666667 | 12.333333 | AG |
| 24050 | N3 | 15.333333 | 10.333333 | 11 | GG |
| 24051 | N4 | 15.333333 | 11 | 12.333333 | GG |
| 24052 | N5 | 15.666667 | 11.333333 | 12.333333 | GG |
| 24053 | N6 | 14.333333 | 8.6666667 | 12 | AG |
| 24054 | N7 | 10.666667 | 4 | 11.333333 | AA |
| 24055 | N8 | 15 | 11.333333 | 11.666667 | AG |
| 24056 | N9 | 4.3333333 | 3 | 8 | AG |
| 24057 | N10 | 15 | 12 | 11.333333 | AG |
| 24058 | N11 | 9 | 7 | 9.6666667 | AG |
| 24059 | N12 | 15.666667 | 10 | 11 | GG |
| 24060 | N13 | 12.333333 | 7.6666667 | 11 | AG |
| 24061 | N14 | 16 | 13.333333 | 12.333333 | GG |
| 24062 | N15 | 15.666667 | 10.666667 | 11.666667 | GG |
| 24063 | N16 | 15.333333 | 10.666667 | 12 | GG |
| 24064 | N17 | 9 | 6.3333333 | 10.666667 | AG |
| 24065 | N18 | 15.666667 | 11 | 12.333333 | GG |
| 24066 | N19 | 16.666667 | 12.666667 | 11.333333 | GG |
| 24067 | N20 | 11.666667 | 8.6666667 | 12 | AG |
| 24068 | N21 | 12.333333 | 8.3333333 | 11.666667 | AG |
| 24069 | N22 | 15.333333 | 11.666667 | 11.666667 | GG |
| 24070 | N23 | 15 | 9.6666667 | 11.666667 | AG |
| 24071 | N24 | 13 | 9.6666667 | 11.333333 | AG |
| 24072 | N25 | 13.666667 | 11.333333 | 12 | AG |
| 24073 | N26 | 15.333333 | 4.6666667 | 11 | GG |
| 24074 | N27 | 14.666667 | 10.333333 | 11.666667 | AG |
| 24075 | N28 | 15.666667 | 13 | 12.333333 | GG |
| 24076 | N29 | 16.666667 | 10.666667 | 10.333333 | GG |
| 24077 | N30 | 15 | 12 | 10.666667 | GG |
| 24078 | N31 | 14.333333 | 8.6666667 | 12 | AG |
| 24079 | N32 | 13.666667 | 7.6666667 | 10 | AG |
| 24080 | N33 | 16 | 10.666667 | 11 | GG |
| 24081 | N34 | 15.666667 | 7 | 10.333333 | GG |
| 24082 | N35 | 15.666667 | 10.666667 | 11.666667 | AG |
| 24083 | N36 | 15.666667 | 12.666667 | 12 | GG |
| 24084 | N37 | 14 | 6.3333333 | 10.666667 | AG |
| 24085 | N38 | 10 | 7.6666667 | 10 | AG |
| 24086 | N39 | 12 | 8.3333333 | 10.666667 | AG |
| 24087 | N40 | 16 | 11.666667 | 11.333333 | GG |
| 24088 | N41 | 0.3333333 | 0 | 0 | AG |
| 24089 | N42 | 9 | 5.3333333 | 8 | AG |
| 24090 | N43 | 14.666667 | 9.6666667 | 12.333333 | AG |
| 24091 | N44 | 15.333333 | 5.6666667 | 10.333333 | GG |
| 24092 | N45 | 14 | 7.6666667 | 11.666667 | AG |
| 24093 | N46 | 6.3333333 | 5 | 8.6666667 | AG |
| 24094 | N47 | 12 | 8 | 11.666667 | AG |
| 24095 | N48 | 14.333333 | 11.333333 | 11.666667 | AG |
| 24096 | O1 | 14 | 8.6666667 | 10 | AG |
| 24097 | O2 | 14.333333 | 7.6666667 | 10.333333 | AG |
| 24098 | O3 | 15 | 9 | 11.666667 | AG |
| 24099 | O4 | 15.666667 | 10 | 11.333333 | GG |
| 24100 | O6 | 16 | 11 | 11.333333 | GG |
| 24101 | O7 | 11.333333 | 9.3333333 | 9.6666667 | GG |
| 24102 | O8 | 15.666667 | 8.6666667 | 11.333333 | GG |
| 24103 | O9 | 13 | 11.333333 | 12 | AG |
| 24104 | O10 | 12.666667 | 8.3333333 | 10.333333 | AG |
| 24105 | O11 | 16.333333 | 13 | 11.666667 | AA |
| 24106 | O12 | 13.666667 | 9.6666667 | 11.333333 | AG |
| 24107 | O13 | 3.6666667 | 2.6666667 | 4 | GG |
| 24108 | O14 | 13.666667 | 8.3333333 | 10.333333 | AG |
| 24109 | O15 | 14 | 10.333333 | 12.333333 | GG |
| 24110 | O16 | 14.666667 | 11 | 11.666667 | AG |
| 24111 | O17 | 14.666667 | 9.3333333 | 11.666667 | GG |
| 24112 | O18 | 13.666667 | 10.666667 | 11.666667 | AG |
| 24113 | O19 | 16 | 12.333333 | 11.666667 | GG |
| 24114 | O20 | 14 | 10 | 11.666667 | AG |
| 24115 | O21 | 11.333333 | 7.3333333 | 9.6666667 | AG |
| 24116 | O22 | 16 | 11 | 10.666667 | AG |
| 24117 | O23 | 13.666667 | 10.666667 | 12 | AG |
| 24118 | O24 | 14 | 11.333333 | 12.333333 | AG |
| 24119 | O25 | 15 | 10 | 11.333333 | AG |
| 24120 | O26 | 14.333333 | 8.3333333 | 11.666667 | AG |
| 24121 | O27 | 14.333333 | 6 | 11.333333 | GG |
| 24122 | O28 | 16 | 8 | 10.333333 | AG |
| 24123 | O29 | 15.333333 | 9.3333333 | 11.666667 | AG |
| 24124 | O30 | 14.333333 | 4.6666667 | 11.333333 | AG |
| 24125 | O31 | 15 | 10.333333 | 11.666667 | AG |
| 24126 | O32 | 15.333333 | 12 | 11.333333 | AG |
| 24127 | O33 | 15.666667 | 10.333333 | 11.333333 | AG |
| 24128 | O34 | 16 | 11.333333 | 12.333333 | AG |
| 24129 | O35 | 16 | 5.6666667 | 10.666667 | AG |
| 24130 | O36 | 12.333333 | 5.6666667 | 11 | AG |
| 24131 | O37 | 0.6666667 | 0 | 0 | AG |
| 24132 | O38 | 14.666667 | 11 | 12 | AA |
| 24133 | O39 | 15 | 11 | 11 | AG |
| 24134 | O40 | 14.333333 | 13 | 12 | AG |
| 24135 | O41 | 15.666667 | 10 | 11.333333 | AG |
| 24136 | O42 | 15 | 9.6666667 | 10.666667 | AG |
| 24137 | O43 | 14.333333 | 7 | 11 | AG |
| 24138 | O44 | 16.333333 | 11.666667 | 11.666667 | AG |
| 24139 | O45 | 10.333333 | 6.6666667 | 11.666667 | GG |
| 24140 | O46 | 15 | 13.333333 | 12 | AG |
| 24141 | O47 | 15.333333 | 9 | 11.333333 | AA |
| 24142 | O48 | 12.666667 | 5.6666667 | 9 | GG |
| 24143 | P1 | 17 | 12.333333 | 12.333333 | AG |
| 24144 | P2 | 16 | 11 | 11 | AG |
| 24145 | P3 | 14.333333 | 11 | 11.666667 | AG |
| 24146 | P4 | 16.666667 | 11 | 11.333333 | AG |
| 24147 | P5 | 15.666667 | 9 | 10.666667 | AG |
| 24148 | P6 | 16.333333 | 9 | 12 | AG |
| 24149 | P7 | 16 | 8.6666667 | 11.333333 | AG |
| 24150 | P8 | 14 | 6 | 11.666667 | AG |
| 24151 | P9 | 15.666667 | 13.333333 | 12.333333 | AG |
| 24152 | P10 | 14.333333 | 9.3333333 | 9.6666667 | AG |
| 24153 | P11 | 16.666667 | 10 | 10.666667 | AG |
| 24154 | P12 | 14 | 9.6666667 | 11.333333 | AG |
| 24155 | P13 | 15.333333 | 11 | 10.333333 | AG |
| 24156 | P14 | 15.333333 | 10.666667 | 12 | GG |
| 24157 | P15 | 15.333333 | 8.6666667 | 9.6666667 | AG |
| 24158 | P16 | 16 | 10.666667 | 11.666667 | AG |
| 24159 | P17 | 14 | 9 | 11.666667 | AG |
| 24160 | P18 | 14 | 9.6666667 | 10.666667 | AG |
| 24161 | P19 | 14.666667 | 10.333333 | 11.666667 | AG |
| 24162 | P20 | 14.666667 | 10 | 11.666667 | AG |
| 24163 | P21 | 15.666667 | 10.666667 | 11 | GG |
| 24164 | P22 | 15.333333 | 12 | 11.666667 | GG |
| 24165 | P23 | 15.666667 | 12.666667 | 11.333333 | AG |
| 24166 | P24 | 14.666667 | 7.6666667 | 11.666667 | AG |
| 24167 | P25 | 15 | 12.666667 | 12 | AG |
| 24168 | P26 | 15.333333 | 9 | 10.666667 | GG |
| 24169 | P27 | 16.333333 | 14 | 15.3333 | AG |
| 24170 | P28 | 12 | 8.3333333 | 11.333333 | AG |
| 24171 | P29 | 16 | 4 | 11.333333 | AG |
| 24172 | P30 | 15 | 11 | 11.666667 | GG |
| 24173 | P31 | 15.333333 | 10.666667 | 11.666667 | GG |
| 24174 | P32 | 16.333333 | 10.333333 | 11.666667 | GG |
| 24175 | P33 | 14.333333 | 12 | 11.666667 | GG |
| 24176 | P34 | 0.3333333 | 0 | 0 | GG |
| 24177 | P35 | 16.666667 | 11.666667 | 11.666667 | AG |
| 24178 | P36 | 14.666667 | 10 | 9.6666667 | AG |
| 24179 | P37 | 15.333333 | 11 | 10.666667 | GG |
| 24180 | P38 | 12.666667 | 6.6666667 | 9.6666667 | AG |
| 24181 | P39 | 16.333333 | 11 | 11 | AG |
| 24182 | P40 | 13.666667 | 9.6666667 | 12.333333 | GG |
| 24183 | P41 | 16.333333 | 6.6666667 | 10.333333 | AG |
| 24184 | P42 | 15.333333 | 9.3333333 | 11 | AG |
| 24185 | P43 | 15 | 9.6666667 | 11.666667 | AG |
| 24186 | P44 | 17 | 7.3333333 | 11.666667 | AG |
| 24187 | P45 | 16.333333 | 11.666667 | 12 | AG |
| 24188 | P46 | 16.333333 | 9.3333333 | 12.333333 | AA |
| 24189 | P47 | 11.666667 | 8.6666667 | 11.666667 | AA |
| 24190 | P48 | 13.666667 | 9.3333333 | 12 | AG |
| 24191 | Q1 | 16.333333 | 9.3333333 | 11.666667 | GG |
| 24192 | Q2 | 14.666667 | 10.666667 | 11.333333 | GG |
| 24193 | Q3 | 16 | 7.3333333 | 11 | GG |
| 24194 | Q4 | 14.666667 | 10.666667 | 11 | AG |
| 24195 | Q5 | 9.6666667 | 5 | 8.6666667 | AG |
| 24196 | Q6 | 7 | 4 | 8.6666667 | AG |
| 24197 | Q7 | 2.6666667 | 0.6666667 | 0 | AG |
| 24198 | Q8 | 10 | 7.3333333 | 11.333333 | AG |
| 24199 | Q9 | 15.333333 | 10 | 11 | AG |
| 24200 | Q10 | 14 | 8.6666667 | 12 | AG |
| 24201 | Q11 | 14.333333 | 7.6666667 | 10.333333 | AG |
| 24202 | Q12 | 6.6666667 | 5.3333333 | 10.666667 | AG |
| 24203 | Q13 | 14 | 11 | 11 | AG |
| 24204 | Q14 | 2.3333333 | 0.6666667 | 0.3333333 | AG |
| 24205 | Q15 | 16 | 7 | 10 | AG |
| 24206 | Q16 | 15.666667 | 12.666667 | 12 | AG |
| 24207 | Q17 | 16.333333 | 6.6666667 | 10.333333 | AG |
| 24208 | Q18 | 16 | 6 | 10.333333 | GG |
| 24209 | Q20 | 13.333333 | 7.6666667 | 10.666667 | GG |
| 24210 | Q21 | 12 | 6.3333333 | 11.333333 | GG |
| 24211 | Q22 | 16.333333 | 10.666667 | 12 | GG |
| 24212 | Q23 | 15 | 9.3333333 | 11.333333 | AG |
| 24213 | Q24 | 17 | 11.666667 | 11.666667 | AG |
| 24214 | Q25 | 15.666667 | 11 | 11.666667 | AG |
| 24215 | Q26 | 15 | 11.666667 | 11.333333 | AG |
| 24216 | Q27 | 17 | 10.333333 | 12 | AG |
| 24217 | Q28 | 16 | 11.666667 | 11 | AG |
| 24218 | Q29 | 13 | 6.6666667 | 10.333333 | AG |
| 24219 | Q30 | 17 | 11.333333 | 11 | AG |
| 24220 | Q31 | 16.666667 | 9 | 11 | GG |
| 24221 | Q32 | 13.666667 | 9.3333333 | 11.333333 | GG |
| 24222 | Q34 | 13 | 7.3333333 | 7.3333333 | GG |
| 24223 | Q35 | 15 | 9 | 12 | GG |
| 24224 | Q36 | 15.333333 | 12 | 11.333333 | AG |
| 24225 | Q39 | 14.666667 | 10 | 11.666667 | AG |
| 24226 | Q40 | 15.333333 | 13 | 12.333333 | AA |
| 24227 | Q42 | 14.666667 | 9.3333333 | 10.333333 | GG |
| 24228 | Q43 | 16.333333 | 7.6666667 | 10 | GG |
| 24229 | Q44 | 14.666667 | 10.666667 | 11 | AG |
| 24230 | Q45 | 9 | 5.3333333 | 9.6666667 | AG |
| 24231 | Q46 | 14.666667 | 9.6666667 | 10.333333 | AG |
| 24232 | Q47 | 16.666667 | 12 | 11.333333 | AG |
| 24233 | Q48 | 14.666667 | 8.6666667 | 12.333333 | AG |
| 24234 | R1 | 15.666667 | 9 | 10.333333 | AG |
| 24235 | R2 | 14.666667 | 11.333333 | 12 | AA |
| 24236 | R3 | 15.333333 | 11 | 12.333333 | AG |
| 24237 | R4 | 15 | 8.6666667 | 10.666667 | AG |
| 24238 | R5 | 12 | 8 | 11.666667 | GG |
| 24239 | R6 | 17 | 11.333333 | 11.666667 | AG |
| 24240 | R7 | 17 | 13 | 12 | GG |
| 24241 | R8 | 16 | 12.666667 | 11.666667 | GG |
| 24242 | R9 | 16 | 8.6666667 | 11.666667 | AG |
| 24243 | R10 | 14.666667 | 8.3333333 | 10.333333 | AG |
| 24244 | R11 | 14.333333 | 10.666667 | 11 | AG |
| 24245 | R12 | 15.666667 | 12.333333 | 10.333333 | AG |
| 24246 | R13 | 13.666667 | 8 | 11 | AG |
| 24247 | R14 | 12.333333 | 5 | 10.333333 | AG |
| 24248 | R15 | 15 | 9.6666667 | 11.666667 | AG |
| 24249 | R16 | 15.666667 | 10.666667 | 11.333333 | AG |
| 24250 | R17 | 15.333333 | 10.666667 | 11.333333 | AG |
| 24251 | R18 | 11.666667 | 7 | 10.666667 | AG |
| 24252 | R19 | 15.333333 | 11.666667 | 11.333333 | AG |
| 24253 | R20 | 17 | 10.666667 | 11.333333 | GG |
| 24254 | R23 | 16 | 12.333333 | 11 | AG |
| 24255 | R24 | 14.333333 | 5.6666667 | 9.6666667 | GG |
| 24256 | R25 | 16.333333 | 12 | 12 | AG |
| 24257 | R28 | 16.333333 | 9.6666667 | 11.666667 | AG |
| 24258 | R30 | 16.666667 | 9 | 11 | AG |
| 24259 | R31 | 14.666667 | 8.6666667 | 11.333333 | AG |
| 24260 | R32 | 14.333333 | 4.3333333 | 11.666667 | AG |
| 24261 | R35 | 16 | 10 | 11.666667 | AG |
| 24262 | R36 | 16.666667 | 9.3333333 | 11.333333 | AG |
| 24263 | R37 | 16.666667 | 7.3333333 | 10.666667 | AG |
| 24264 | R38 | 14.666667 | 11.666667 | 11.333333 | GG |
| 24265 | R39 | 15.666667 | 11 | 11.333333 | GG |
| 24266 | R40 | 14.333333 | 11 | 11 | GG |
| 24267 | R41 | 14.333333 | 9 | 11.333333 | AG |
| 24268 | R42 | 16 | 9 | 11.333333 | GG |
| 24269 | R43 | 12.666667 | 8 | 12 | AA |
| 24270 | R44 | 9.3333333 | 5.6666667 | 8.6666667 | AG |
| 24271 | R46 | 15 | 10 | 11.666667 | AG |
| 24272 | R47 | 15 | 12.333333 | 11.666667 | AG |
| 24273 | R48 | 14.666667 | 9.3333333 | 12.333333 | AG |
| 24274 | Q19 | 16.666667 | 7.3333333 | 11.333333 | AG |
| 24275 | Q37 | 16 | 7.3333333 | 10 | AG |
| 24276 | Q38 | 14.666667 | 12 | 12 | AG |
| 24277 | Q41 | 15.666667 | 10 | 11.666667 | AG |
| 24278 | R22 | 12 | 10 | 10.666667 | AG |
| 24279 | R29 | 11.666667 | 8 | 11 | AA |
| 24280 | R21 | 11 | 9.3333333 | 11.333333 | AG |
| 24281 | R26 | 13.666667 | 6 | 11 | AG |
| 24282 | R27 | 11.666667 | 6.3333333 | 11 | AG |
| 24283 | R34 | 15.666667 | 9 | 11.666667 | AG |
| 24284 | S1 | 14.333333 | 8.3333333 | 11 | AA |
| 24285 | S2 | 17 | 8 | 11 | AG |
| 24286 | S3 | 14 | 9 | 11.666667 | GG |
| 24287 | S4 | 16 | 10.666667 | 12 | GG |
| 24288 | S5 | 15.333333 | 8.3333333 | 11.333333 | AG |
| 24289 | S6 | 16 | 11.333333 | 10.666667 | AG |
| 24290 | S7 | 15.666667 | 10.333333 | 11 | AG |
| 24291 | S8 | 14.666667 | 8.6666667 | 9.6666667 | AG |
| 24292 | S9 | 15.666667 | 10.333333 | 11.333333 | AG |
| 24293 | S10 | 1 | 0.6666667 | 0.6666667 | AG |
| 24294 | S11 | 15 | 10.333333 | 11.666667 | AG |
| 24295 | S12 | 12.666667 | 7.6666667 | 11.666667 | AG |
| 24296 | S13 | 17 | 10.333333 | 11.333333 | GG |
| 24297 | S14 | 13.666667 | 8 | 11.333333 | AA |
| 24298 | S15 | 17 | 11.333333 | 11.666667 | GG |
| 24299 | S16 | 0.6666667 | 0.6666667 | 3.6666667 | GG |
| 24300 | S17 | 12 | 9 | 11.333333 | GG |
| 24301 | S18 | 14 | 10.333333 | 12 | AG |
| 24302 | S19 | 13.666667 | 8.6666667 | 11.333333 | GG |
| 24303 | S20 | 15 | 10.666667 | 11 | GG |
| 24304 | S21 | 16.666667 | 10 | 9.6666667 | GG |
| 24305 | S22 | 11 | 4.3333333 | 9.6666667 | AA |
| 24306 | S23 | 9 | 6.6666667 | 10.333333 | AG |
| 24307 | S24 | 15 | 10 | 11.333333 | AG |
| 24308 | S25 | 16 | 8.3333333 | 10 | GG |
| 24309 | S26 | 15.666667 | 8 | 11.333333 | AA |
| 24310 | S27 | 14 | 7.3333333 | 10.666667 | GG |
| 24311 | S28 | 14.666667 | 10 | 10.333333 | GG |
| 24312 | S29 | 14.666667 | 9.6666667 | 11.333333 | GG |
| 24313 | S30 | 16.333333 | 9 | 11.666667 | AG |
| 24314 | S31 | 15.666667 | 11.666667 | 12 | GG |
| 24315 | S32 | 15.666667 | 10.333333 | 11 | GG |
| 24316 | S33 | 16 | 12 | 11.333333 | GG |
| 24317 | S34 | 14.666667 | 8.3333333 | 10.333333 | AA |
| 24318 | S35 | 13 | 8.6666667 | 11 | AG |
| 24319 | S36 | 12.666667 | 11 | 11.666667 | AG |
| 24320 | S37 | 15.666667 | 9.6666667 | 9.3333333 | GG |
| 24321 | S38 | 15 | 11 | 11.666667 | GG |
| 24322 | S39 | 14.666667 | 7.6666667 | 11.333333 | GG |
| 24323 | S40 | 15.333333 | 12.333333 | 11.666667 | GG |
| 24324 | S41 | 16.333333 | 10 | 11.666667 | AG |
| 24325 | S42 | 16 | 11.333333 | 11.666667 | GG |
| 24326 | S43 | 16 | 12 | 11.666667 | GG |
| 24327 | S44 | 15 | 8.3333333 | 11 | AG |
| 24328 | S45 | 15.333333 | 9.6666667 | 11 | AG |
| 24329 | S46 | 15 | 13.333333 | 12.333333 | AG |
| 24330 | S47 | 13 | 9.3333333 | 12 | GG |
| 24331 | S48 | 13.333333 | 9 | 12.333333 | GG |
| 24332 | T1 | 15 | 12 | 11.333333 | AA |
| 24333 | T3 | 15 | 10.666667 | 9.6666667 | AG |
| 24334 | T4 | 13.333333 | 6.3333333 | 10 | AG |
| 24335 | T5 | 14 | 3.6666667 | 11.333333 | AG |
| 24336 | T6 | 12.333333 | 8 | 11.333333 | AG |
| 24337 | T7 | 15.666667 | 7.3333333 | 10 | AG |
| 24338 | T8 | 4.3333333 | 2.3333333 | 9 | AG |
| 24339 | T9 | 15.333333 | 12 | 11.666667 | GG |
| 24340 | T10 | 13.666667 | 11.666667 | 11.666667 | GG |
| 24341 | T11 | 16.666667 | 13.666667 | 14.3333 | AA |
| 24342 | T12 | 14.333333 | 11.333333 | 11.333333 | AA |
| 24343 | T13 | 14.333333 | 10.333333 | 11.333333 | AG |
| 24344 | T14 | 15.333333 | 10.333333 | 11.666667 | GG |
| 24345 | T15 | 15.333333 | 10.333333 | 10.666667 | AG |
| 24346 | T16 | 10.333333 | 7 | 9.3333333 | AG |
| 24347 | T17 | 16 | 11 | 11.666667 | AG |
| 24348 | T18 | 16.666667 | 12.666667 | 11.333333 | AG |
| 24349 | T19 | 8.6666667 | 5.3333333 | 10 | AA |
| 24350 | T20 | 12.333333 | 8 | 10.333333 | AG |
| 24351 | T21 | 14.666667 | 9.6666667 | 10.666667 | GG |
| 24352 | T22 | 14.666667 | 7.3333333 | 11 | AG |
| 24353 | T23 | 13.666667 | 10.666667 | 11 | GG |
| 24354 | T24 | 15.666667 | 12 | 11.333333 | AG |
| 24355 | T25 | 13.666667 | 9.3333333 | 11.333333 | AG |
| 24356 | T26 | 16.333333 | 14 | 16.3333 | AG |
| 24357 | T27 | 11.333333 | 5 | 8.3333333 | AG |
| 24358 | T28 | 15 | 8.6666667 | 11.333333 | AG |
| 24359 | T29 | 14.666667 | 10 | 11.333333 | AG |
| 24360 | T30 | 17 | 7.6666667 | 11.666667 | AG |
| 24361 | T31 | 16.333333 | 9.6666667 | 11.666667 | AG |
| 24362 | T32 | 14.333333 | 9.3333333 | 12.333333 | AG |
| 24363 | T33 | 14 | 8.6666667 | 11 | GG |
| 24364 | T34 | 14.666667 | 9.6666667 | 10.666667 | AG |
| 24365 | T35 | 15 | 12 | 12.333333 | GG |
| 24366 | T36 | 13.666667 | 10.666667 | 11.666667 | AG |
| 24367 | T37 | 7.6666667 | 3.6666667 | 6.6666667 | GG |
| 24368 | T38 | 16.666667 | 11.666667 | 11.333333 | GG |
| 24369 | T39 | 15.666667 | 11.333333 | 11 | GG |
| 24370 | T40 | 10.666667 | 8 | 11.666667 | GG |
| 24371 | T41 | 15.333333 | 9.6666667 | 11.333333 | AG |
| 24372 | T42 | 15 | 9.6666667 | 11 | AA |
| 24373 | T43 | 15.666667 | 9 | 11.666667 | GG |
| 24374 | T44 | 15 | 11 | 10.666667 | GG |
| 24375 | T45 | 15.333333 | 6.6666667 | 11.666667 | AA |
| 24376 | T46 | 14.333333 | 9.3333333 | 11 | GG |
| 24377 | T47 | 13.666667 | 8.6666667 | 10.333333 | GG |
| 24378 | T48 | 13 | 9 | 11.333333 | AG |
| 24379 | U1 | 15.666667 | 9 | 10.333333 | AG |
| 24380 | U2 | 17 | 10.333333 | 12 | GG |
| 24381 | U3 | 16 | 8.6666667 | 11.333333 | AA |
| 24382 | U4 | 3 | 2 | 4.3333333 | AA |
| 24383 | U5 | 16.666667 | 11 | 12.333333 | GG |
| 24384 | U6 | 16.666667 | 13.666667 | 16 | GG |
| 24385 | U7 | 16 | 14.333333 | 16 | AA |
| 24386 | U8 | 14.666667 | 9.3333333 | 11.333333 | GG |
| 24387 | U9 | 16.333333 | 10.666667 | 11.666667 | GG |
| 24388 | U10 | 15.333333 | 10.666667 | 11 | GG |
| 24389 | U12 | 15.666667 | 12 | 11.333333 | GG |
| 24390 | U13 | 5 | 2.6666667 | 5.3333333 | AA |
| 24391 | U14 | 7.6666667 | 5.6666667 | 10.666667 | GG |
| 24392 | U15 | 12 | 6.6666667 | 11 | GG |
| 24393 | U16 | 16.666667 | 12.333333 | 11.666667 | AG |
| 24394 | U17 | 15.333333 | 8 | 10.333333 | GG |
| 24395 | U18 | 10 | 6 | 10.666667 | GG |
| 24396 | U19 | 14.666667 | 10 | 11.666667 | GG |
| 24397 | U20 | 15.333333 | 11.333333 | 11 | GG |
| 24398 | U21 | 15.333333 | 8.3333333 | 10.333333 | GG |
| 24399 | U22 | 15 | 8.3333333 | 10 | GG |
| 24400 | U23 | 13.333333 | 7.6666667 | 10.666667 | GG |
| 24401 | U24 | 13.666667 | 11 | 12 | GG |
| 24402 | U25 | 15 | 10.666667 | 11 | GG |
| 24403 | U26 | 14.333333 | 7.3333333 | 10.333333 | AA |
| 24404 | U27 | 15.333333 | 11.333333 | 11 | GG |
| 24405 | U28 | 16 | 12.666667 | 11.333333 | GG |
| 24406 | U29 | 13.666667 | 9.6666667 | 10.666667 | GG |
| 24407 | U30 | 13.333333 | 6 | 11 | GG |
| 24408 | U31 | 15.333333 | 12 | 12 | GG |
| 24409 | U32 | 16 | 8 | 10.666667 | GG |
| 24410 | U33 | 13.333333 | 10 | 11.666667 | GG |
| 24411 | U34 | 14.333333 | 10.333333 | 11 | GG |
| 24412 | U35 | 14.333333 | 8.3333333 | 9.6666667 | GG |
| 24413 | U36 | 16.666667 | 12.333333 | 11.333333 | GG |
| 24414 | U37 | 16.666667 | 12 | 10.666667 | GG |
| 24415 | U38 | 15.333333 | 12 | 12.333333 | GG |
| 24416 | U39 | 16.666667 | 11 | 11.666667 | AA |
| 24417 | U40 | 12.666667 | 8.3333333 | 11.333333 | AG |
| 24418 | U41 | 3 | 2.6666667 | 5.6666667 | AG |
| 24419 | U42 | 13.666667 | 5.6666667 | 10 | AG |
| 24420 | U43 | 15.666667 | 12.333333 | 11.666667 | AG |
| 24421 | U44 | 16.666667 | 10.666667 | 11.666667 | AA |
| 24422 | U45 | 9.6666667 | 6.3333333 | 8.3333333 | AA |
| 24423 | U46 | 13 | 8.6666667 | 11.666667 | AG |
| 24424 | U47 | 13 | 10 | 11.666667 | AG |
| 24425 | U48 | 14.666667 | 11 | 11 | AG |
| 24426 | V1 | 14.666667 | 8 | 12 | GG |
| 24427 | V2 | 16.333333 | 8.6666667 | 8.6666667 | AA |
| 24428 | V3 | 16.333333 | 10 | 11.666667 | GG |
| 24429 | V4 | 12.666667 | 7.3333333 | 10.666667 | AG |
| 24430 | V5 | 14 | 4.6666667 | 9 | AG |
| 24431 | V6 | 16 | 7.6666667 | 11.333333 | AG |
| 24432 | V7 | 12.333333 | 9 | 9.3333333 | AG |
| 24433 | V8 | 16 | 9.6666667 | 11.666667 | AG |
| 24434 | V9 | 12.666667 | 8.6666667 | 10 | AG |
| 24435 | V10 | 14.666667 | 10 | 12 | GG |
| 24436 | V11 | 12 | 9.3333333 | 10.333333 | GG |
| 24437 | V12 | 15 | 10.333333 | 10.333333 | GG |
| 24438 | V13 | 14 | 10.666667 | 11.333333 | GG |
| 24439 | V14 | 14 | 11 | 12 | GG |
| 24440 | V15 | 4.3333333 | 2 | 3.3333333 | GG |
| 24441 | V16 | 14.333333 | 11.333333 | 11.333333 | AA |
| 24442 | V17 | 14.333333 | 10.666667 | 12.333333 | GG |
| 24443 | V18 | 8.3333333 | 5 | 6.6666667 | AG |
| 24444 | V19 | 15.666667 | 7.6666667 | 11.666667 | AG |
| 24445 | V20 | 10.333333 | 6.3333333 | 9 | GG |
| 24446 | V21 | 11.333333 | 7.3333333 | 11.666667 | GG |
| 24447 | V22 | 14 | 11.333333 | 12 | GG |
| 24448 | V23 | 16 | 10 | 12 | GG |
| 24449 | V24 | 16 | 9.6666667 | 11.333333 | AA |
| 24450 | V25 | 16 | 14.333333 | 16 | GG |
| 24451 | V26 | 14.666667 | 9.6666667 | 11.333333 | GG |
| 24452 | V27 | 9.3333333 | 6.3333333 | 8.6666667 | GG |
| 24453 | V28 | 14.666667 | 7.6666667 | 11.333333 | AG |
| 24454 | V29 | 16 | 11.333333 | 11.333333 | GG |
| 24455 | V30 | 16.666667 | 11.666667 | 12.333333 | GG |
| 24456 | V31 | 6.6666667 | 5.6666667 | 8.6666667 | AA |
| 24457 | V32 | 13.666667 | 9.3333333 | 11.666667 | AA |
| 24458 | V33 | 14.666667 | 8.6666667 | 11.333333 | GG |
| 24459 | V34 | 13.666667 | 10.333333 | 11.666667 | AA |
| 24460 | V35 | 14.333333 | 10 | 11.333333 | GG |
| 24461 | V36 | 4.6666667 | 3 | 8.3333333 | GG |
| 24462 | V37 | 11.666667 | 4.6666667 | 9.6666667 | GG |
| 24463 | V38 | 16 | 11 | 12 | GG |
| 24464 | V39 | 15.333333 | 9.3333333 | 11 | GG |
| 24465 | V40 | 16 | 10 | 11.333333 | GG |
| 24466 | V41 | 14.333333 | 11 | 11.666667 | GG |
| 24467 | V42 | 11.333333 | 6.3333333 | 11.333333 | AA |
| 24468 | V43 | 15 | 12.333333 | 12 | GG |
| 24469 | V44 | 11.666667 | 6.6666667 | 11.666667 | AA |
| 24470 | V45 | 9.3333333 | 6.6666667 | 8 | AA |
| 24471 | V46 | 15.333333 | 13 | 12 | GG |
| 24472 | V47 | 16.666667 | 12 | 12.333333 | GG |
| 24473 | V48 | 13.666667 | 7.6666667 | 11.666667 | AA |

**Notes:** CAGE (cage number); wg_no (wing number); EN (egg number); FN (the number of fertile eggs after a single AI); DN (the number of days post-insemination until last fertile egg).

**Raw data of association at *lncRNA8138.1* gene.**

| **P2-Wg_no** | **Avg. EN** | **Avg. FN** | **Avg. DN** | **(A/G) mutation** |
| --- | --- | --- | --- | --- |
| ID1 | 12 | 8.33333333 | 7 | GG |
| ID2 | 15.66666667 | 4 | 12.33333 | AA |
| ID3 | 15.33333333 | 11.8333333 | 10.66667 | AA |
| ID4 | 13 | 11.3333333 | 11 | GA |
| ID5 | 14 | 12.8333333 | 13 | GG |
| ID6 | 15.66666667 | 11.5 | 10 | GA |
| ID7 | 13 | 11.6666667 | 15 | GA |
| ID8 | 15 | 12.6666667 | 13 | GA |
| ID9 | 14.66666667 | 12 | 10 | GG |
| ID10 | 13.66666667 | 8.33333333 | 7 | GA |
| ID11 | 14.66666667 | 10.3333333 | 10.66667 | GA |
| ID12 | 14.66666667 | 10.3333333 | 11 | GG |
| ID13 | 14 | 8 | 8.333333 | AA |
| ID14 | 15.66666667 | 14 | 12.66667 | AA |
| ID15 | 15 | 11.3333333 | 9.333333 | AA |
| ID16 | 13.66666667 | 11.8333333 | 13.33333 | GA |
| ID17 | 9.666666667 | 5 | 11.33333 | AA |
| ID18 | 8.333333333 | 6.66666667 | 6 | GA |
| ID19 | 15.33333333 | 4 | 11 | AA |
| ID20 | 14.33333333 | 9 | 10 | GA |
| ID21 | 14.33333333 | 11.3333333 | 11.66667 | GA |
| ID22 | 15 | 10.3333333 | 9.333333 | GA |
| ID23 | 15.66666667 | 11 | 11 | GA |
| ID24 | 13 | 11.6666667 | 11.66667 | GG |
| ID25 | 15 | 12.6666667 | 11.66667 | GA |
| ID26 | 12 | 12.6666667 | 16 | GA |
| ID27 | 16 | 12 | 12.33333 | GA |
| ID28 | 16 | 11.3333333 | 12.33333 | GA |
| ID29 | 15 | 3 | 10.66667 | AA |
| ID30 | 14 | 10.6666667 | 11.66667 | GA |
| ID31 | 15.33333333 | 6 | 13 | AA |
| ID32 | 16 | 10.1666667 | 10 | GA |
| ID33 | 14 | 13.3333333 | 13 | GA |
| ID34 | 15 | 12 | 12.33333 | GA |
| ID35 | 15.33333333 | 13 | 11.66667 | AA |
| ID36 | 15.33333333 | 9.66666667 | 9.333333 | GA |
| ID37 | 14.66666667 | 6 | 12 | AA |
| ID38 | 14.66666667 | 10.6666667 | 11 | GA |
| ID39 | 15.66666667 | 10.1666667 | 8 | GA |
| ID40 | 16.33333333 | 14 | 13.33333 | AA |
| ID41 | 15.66666667 | 13.3333333 | 14.33333 | GA |
| ID42 | 14.33333333 | 6 | 12.33333 | AA |
| ID43 | 15.66666667 | 13.6666667 | 12 | GA |
| ID44 | 15.33333333 | 6 | 11 | AA |
| ID45 | 15.66666667 | 12.3333333 | 12 | GA |
| ID46 | 15.33333333 | 4 | 11.33333 | AA |
| ID47 | 15.66666667 | 11 | 10.33333 | GA |
| ID48 | 15 | 9.66666667 | 9 | GA |
| ID49 | 15.33333333 | 12 | 11.66667 | GA |
| ID50 | 16 | 11.3333333 | 11.33333 | AA |
| ID51 | 14.66666667 | 9 | 8.333333 | GA |
| ID52 | 16.33333333 | 10.6666667 | 9.666667 | AA |
| ID53 | 16.66666667 | 14.6666667 | 13.66667 | GA |
| ID54 | 14.66666667 | 10.3333333 | 10.33333 | GA |
| ID55 | 14 | 8.33333333 | 8 | AA |
| ID56 | 15.66666667 | 10.3333333 | 11 | GA |
| ID57 | 14.33333333 | 10.6666667 | 11 | GA |
| ID58 | 14.66666667 | 10 | 10.66667 | GA |
| ID59 | 14.66666667 | 10 | 10.66667 | GA |
| ID60 | 12.33333333 | 6 | 11.66667 | AA |
| ID61 | 13.66666667 | 9 | 8.666667 | GA |
| ID62 | 12.66666667 | 9 | 10 | GA |
| ID63 | 16 | 11.3333333 | 11 | GA |
| ID64 | 14.33333333 | 10.6666667 | 10 | GA |
| ID65 | 15.66666667 | 13.3333333 | 13.33333 | GA |
| ID66 | 14.66666667 | 11 | 9.333333 | AA |
| ID67 | 14.33333333 | 10.3333333 | 11.33333 | GA |
| ID68 | 16.33333333 | 8.66666667 | 8 | GA |
| ID69 | 13.66666667 | 12 | 11 | GA |
| ID70 | 12.33333333 | 11 | 12.66667 | GA |
| ID71 | 16 | 12.6666667 | 11.33333 | AA |
| ID72 | 15.66666667 | 13.6666667 | 15.33333 | GG |
| ID73 | 15 | 9.33333333 | 7.333333 | AA |
| ID74 | 16.33333333 | 14 | 14 | GA |
| ID75 | 15 | 12 | 11.66667 | GA |
| ID76 | 14 | 9 | 8 | AA |
| ID77 | 16.33333333 | 11 | 10.33333 | GA |
| ID78 | 15.66666667 | 10 | 8.333333 | AA |
| ID79 | 16.33333333 | 13 | 12.66667 | GA |
| ID80 | 14.66666667 | 10.3333333 | 10.33333 | GA |
| ID81 | 15 | 13 | 12 | GA |
| ID82 | 14.66666667 | 10.6666667 | 9.333333 | GG |
| ID83 | 14.33333333 | 10.6666667 | 10.66667 | GG |
| ID84 | 16.66666667 | 12.3333333 | 9.333333 | AA |
| ID85 | 17 | 11.6666667 | 11 | GA |
| ID86 | 15 | 11 | 10.33333 | GA |
| ID87 | 14.33333333 | 12.6666667 | 12 | GG |
| ID88 | 15.33333333 | 10.6666667 | 9.333333 | GG |
| ID89 | 14 | 6 | 11 | AA |
| ID90 | 11.66666667 | 10 | 9.333333 | GA |
| ID91 | 15.66666667 | 14.6666667 | 14.66667 | GA |
| ID92 | 12.33333333 | 11.6666667 | 11 | GA |
| ID93 | 14 | 12.3333333 | 13.33333 | GA |
| ID94 | 15.66666667 | 12 | 13 | GA |
| ID95 | 14.33333333 | 11.6666667 | 12.33333 | AA |
| ID96 | 15.66666667 | 14 | 15.33333 | AA |
| ID97 | 13.66666667 | 10.1666667 | 12.33333 | GA |
| ID98 | 14.33333333 | 11 | 12 | AA |
| ID99 | 16 | 11.6666667 | 10.33333 | GA |
| ID100 | 15.66666667 | 10.3333333 | 9.666667 | GA |
| ID101 | 14 | 10.1666667 | 10.66667 | GA |
| ID102 | 15.33333333 | 12 | 11.33333 | GA |
| ID103 | 15.66666667 | 10.6666667 | 8 | GA |
| ID104 | 13 | 13.3333333 | 16 | GA |
| ID105 | 12.66666667 | 11 | 10.66667 | GA |
| ID106 | 15.66666667 | 10.3333333 | 8.666667 | GA |
| ID107 | 14.33333333 | 14 | 13 | GA |
| ID108 | 12 | 11.3333333 | 12 | GA |
| ID109 | 13.33333333 | 11 | 10.66667 | GA |
| ID110 | 15 | 12.6666667 | 10 | GA |
| ID111 | 14.33333333 | 11 | 10 | GA |
| ID112 | 14.66666667 | 9.66666667 | 11.66667 | GA |
| ID113 | 14 | 11.6666667 | 12.33333 | AA |
| ID114 | 13 | 14.3333333 | 16.66667 | GA |
| ID115 | 14.33333333 | 11 | 10 | GA |
| ID116 | 14 | 12 | 11 | AA |
| ID117 | 15.66666667 | 9 | 7.666667 | AA |
| ID118 | 13 | 11.3333333 | 13.66667 | GA |
| ID119 | 15.33333333 | 11.1666667 | 10.66667 | GA |
| ID120 | 14.66666667 | 12.3333333 | 13.66667 | GA |
| ID121 | 14 | 9.33333333 | 9 | AA |
| ID122 | 16.33333333 | 12.6666667 | 11.33333 | GA |
| ID123 | 16.33333333 | 10.3333333 | 9.333333 | GA |
| ID124 | 15.33333333 | 12.3333333 | 10 | GA |
| ID125 | 14.33333333 | 9.33333333 | 9.666667 | GA |
| ID126 | 14.66666667 | 11.6666667 | 11.33333 | GA |
| ID127 | 14.66666667 | 14 | 15 | GA |
| ID128 | 14 | 13 | 15.33333 | GA |
| ID129 | 11.33333333 | 10 | 12 | AA |
| ID130 | 16 | 14 | 13.66667 | AA |
| ID131 | 13.66666667 | 10 | 9.333333 | GA |
| ID132 | 15 | 10 | 11.33333 | GA |
| ID133 | 13.33333333 | 10 | 8.666667 | GA |
| ID134 | 15.33333333 | 11.3333333 | 10 | GA |
| ID135 | 16 | 11 | 11 | GA |
| ID136 | 13.66666667 | 10.3333333 | 8.666667 | GA |
| ID137 | 16.66666667 | 6 | 9.666667 | AA |
| ID138 | 14.66666667 | 9.16666667 | 8 | GA |
| ID139 | 15.66666667 | 10.3333333 | 10.33333 | GA |
| ID140 | 14.33333333 | 10.3333333 | 9 | GA |
| ID141 | 15 | 11.3333333 | 11.66667 | GA |
| ID142 | 13 | 11 | 11.33333 | AA |
| ID143 | 14 | 13 | 13.66667 | GA |
| ID144 | 16.33333333 | 13.3333333 | 10.66667 | GA |
| ID145 | 15.33333333 | 12.3333333 | 11.66667 | GA |
| ID146 | 14 | 10 | 7 | GA |
| ID147 | 16.33333333 | 8.33333333 | 7 | GA |
| ID148 | 14.66666667 | 12.1666667 | 10.33333 | GA |
| ID149 | 15 | 12.6666667 | 15 | GA |
| ID150 | 10.33333333 | 8.66666667 | 7.333333 | GA |
| ID151 | 13.66666667 | 10.6666667 | 11.33333 | GA |
| ID152 | 14 | 10 | 9.333333 | GA |
| ID153 | 15.33333333 | 10.6666667 | 9.666667 | GA |
| ID154 | 16.66666667 | 14 | 12.66667 | AA |
| ID155 | 15.33333333 | 10 | 10.33333 | AA |
| ID156 | 14.66666667 | 13.6666667 | 14.33333 | GA |
| ID157 | 13 | 12.6666667 | 13 | GA |
| ID158 | 16.66666667 | 12.6666667 | 11.33333 | AA |
| ID159 | 17 | 14 | 13.66667 | AA |
| ID160 | 14.66666667 | 14 | 13.33333 | GA |
| ID161 | 14.33333333 | 9.33333333 | 10.33333 | GA |
| ID162 | 15.66666667 | 13.3333333 | 14 | GA |
| ID163 | 15 | 10.3333333 | 9 | AA |
| ID164 | 15.33333333 | 10.6666667 | 9.333333 | GA |
| ID165 | 12 | 12 | 12.66667 | AA |
| ID166 | 15.66666667 | 8.66666667 | 6.666667 | AA |
| ID167 | 14.66666667 | 14 | 12.66667 | GA |
| ID168 | 16 | 12 | 9 | GA |
| ID169 | 14 | 8 | 7.333333 | GA |
| ID170 | 14.66666667 | 10.3333333 | 8 | GA |
| ID171 | 4 | 9.33333333 | 10.66667 | AA |
| ID172 | 15 | 11.8333333 | 12.33333 | GA |
| ID173 | 14 | 9.33333333 | 7.333333 | GA |
| ID174 | 14.66666667 | 10.3333333 | 11.66667 | GA |
| ID175 | 1 | 6.33333333 | 8.666667 | GA |
| ID176 | 14.66666667 | 13.5 | 14.66667 | GA |
| ID177 | 14.66666667 | 11.1666667 | 13.66667 | GA |
| ID178 | 14.33333333 | 12 | 12 | GA |
| ID179 | 13 | 12.3333333 | 14 | GA |
| ID180 | 14.66666667 | 12.3333333 | 12.33333 | GA |
| ID181 | 15.33333333 | 6 | 12.66667 | AA |
| ID182 | 15.33333333 | 12.3333333 | 10.33333 | GA |
| ID183 | 15.66666667 | 12.3333333 | 12 | GA |
| ID184 | 16 | 13 | 13 | GA |
| ID185 | 14.66666667 | 12 | 10 | AA |
| ID186 | 14.66666667 | 11.3333333 | 9.333333 | GA |
| ID187 | 17 | 9 | 11.66667 | GA |
| ID188 | 12.33333333 | 6 | 9.333333 | AA |
| ID189 | 17.33333333 | 6.66666667 | 11.66667 | GA |
| ID190 | 16 | 12.3333333 | 16 | GA |
| ID191 | 17 | 13 | 13 | GA |
| ID192 | 16.33333333 | 9 | 10.33333 | GA |
| ID193 | 13.5 | 13.3333333 | 15 | GA |
| ID194 | 17.33333333 | 10.6666667 | 13 | AA |
| ID195 | 15 | 9 | 12.33333 | GA |
| ID196 | 16.66666667 | 9.66666667 | 12 | GA |
| ID197 | 12.33333333 | 11 | 18 | AA |
| ID198 | 16.66666667 | 13.6666667 | 16 | GA |
| ID199 | 17.33333333 | 11.1666667 | 14.33333 | AA |
| ID200 | 15 | 7.66666667 | 11.66667 | GA |
| ID201 | 18.33333333 | 10.6666667 | 13 | GA |
| ID202 | 16.66666667 | 10 | 14 | GA |
| ID203 | 15 | 2.16666667 | 4.333333 | AA |
| ID204 | 17.66666667 | 4 | 7.333333 | GA |
| ID205 | 19.66666667 | 13.3333333 | 13.66667 | GA |
| ID206 | 16.33333333 | 11.1666667 | 13.66667 | AA |
| ID207 | 16.33333333 | 2.66666667 | 3.666667 | GA |
| ID208 | 16.66666667 | 9.66666667 | 14 | GA |
| ID209 | 15 | 7.66666667 | 10.33333 | AA |
| ID210 | 17.66666667 | 10 | 12.66667 | GA |
| ID211 | 18 | 10.1666667 | 13 | GA |
| ID212 | 17 | 10.3333333 | 13.66667 | GA |
| ID213 | 19 | 13.6666667 | 13.66667 | AA |
| ID214 | 15 | 9.33333333 | 13.33333 | GA |
| ID215 | 0 | 0 | 0 | AA |
| ID216 | 12 | 11.3333333 | 12.33333 | AA |
| ID217 | 13.33333333 | 8.66666667 | 13.66667 | GG |
| ID218 | 17.33333333 | 10.1666667 | 11.66667 | GA |
| ID219 | 16 | 10.3333333 | 13.66667 | GG |
| ID220 | 16.33333333 | 4 | 9.666667 | AA |
| ID221 | 19 | 10 | 11.33333 | GA |
| ID222 | 17.33333333 | 9.33333333 | 11 | GA |
| ID223 | 19 | 10.6666667 | 12 | GA |
| ID224 | 17.66666667 | 11.1666667 | 14.33333 | GA |
| ID225 | 16 | 11.6666667 | 14.33333 | AA |
| ID226 | 16.66666667 | 6.66666667 | 6.666667 | GA |
| ID227 | 16 | 7 | 10.66667 | GA |
| ID228 | 15 | 8.66666667 | 12 | GA |
| ID229 | 10.66666667 | 6.66666667 | 8.666667 | GA |
| ID230 | 12.33333333 | 7.33333333 | 11.66667 | GA |
| ID231 | 18 | 11 | 12.66667 | GA |
| ID232 | 16 | 3 | 12.66667 | GA |
| ID233 | 14.66666667 | 10.3333333 | 14 | AA |
| ID234 | 16.66666667 | 9.66666667 | 12.33333 | AA |
| ID235 | 14 | 10 | 13.66667 | GA |
| ID236 | 18.33333333 | 9.66666667 | 11.66667 | GA |
| ID237 | 9.666666667 | 4.66666667 | 8 | GA |
| ID238 | 16.66666667 | 2.66666667 | 10 | GA |
| ID239 | 16.33333333 | 2.33333333 | 11.33333 | GA |
| ID240 | 12.33333333 | 7.16666667 | 12.66667 | GA |
| ID241 | 15.66666667 | 10 | 12.33333 | AA |
| ID242 | 14.66666667 | 6.66666667 | 8.666667 | GG |
| ID243 | 15.33333333 | 8 | 13.33333 | GA |
| ID244 | 8.333333333 | 7.33333333 | 8.666667 | GA |
| ID245 | 14.33333333 | 7 | 12 | GA |
| ID246 | 17.33333333 | 9.33333333 | 12.66667 | GA |
| ID247 | 19 | 11.6666667 | 11.33333 | GA |
| ID248 | 13 | 6 | 8.666667 | GA |
| ID249 | 19.66666667 | 11.5 | 14 | GA |
| ID250 | 19.33333333 | 8.66666667 | 9.666667 | AA |
| ID251 | 13.33333333 | 9.16666667 | 14 | GA |
| ID252 | 16.66666667 | 7.33333333 | 9.333333 | GA |
| ID253 | 14 | 9 | 13 | GA |
| ID254 | 17.33333333 | 7.33333333 | 8.666667 | GG |
| ID255 | 18.33333333 | 12 | 13.33333 | GA |
| ID256 | 18 | 3 | 3 | AA |
| ID257 | 17 | 11.6666667 | 14.33333 | AA |
| ID258 | 19.33333333 | 9.33333333 | 9.333333 | AA |
| ID259 | 17.33333333 | 12 | 13.66667 | GA |
| ID260 | 15.66666667 | 12 | 12.66667 | GA |
| ID261 | 14 | 6.66666667 | 9.666667 | GA |
| ID262 | 15.66666667 | 13.3333333 | 14.33333 | GA |
| ID263 | 16 | 8 | 12 | GA |
| ID264 | 19.66666667 | 2 | 9.333333 | GA |
| ID265 | 16.66666667 | 10.6666667 | 13.33333 | GA |
| ID266 | 16.33333333 | 8.16666667 | 12.66667 | GA |
| ID267 | 17.66666667 | 11 | 13 | GA |
| ID268 | 17 | 8.66666667 | 11.66667 | GG |
| ID269 | 16.33333333 | 10.6666667 | 13.66667 | GA |
| ID270 | 16.33333333 | 10.6666667 | 13.33333 | GA |
| ID271 | 14.66666667 | 8.33333333 | 11.33333 | GA |
| ID272 | 16.33333333 | 8.83333333 | 13 | GG |
| ID273 | 18.33333333 | 11.6666667 | 12 | GG |
| ID274 | 17.33333333 | 9.83333333 | 12.33333 | GA |
| ID275 | 16.66666667 | 5.33333333 | 10 | GG |
| ID276 | 15 | 2.66666667 | 10.33333 | GA |
| ID277 | 19.33333333 | 13.6666667 | 13.66667 | GG |
| ID278 | 17.33333333 | 9 | 10 | GA |
| ID279 | 17.66666667 | 13 | 14 | GA |
| ID280 | 16.33333333 | 2 | 11.33333 | GG |
| ID281 | 15.66666667 | 9.16666667 | 8.666667 | GG |
| ID282 | 14.66666667 | 9 | 13.66667 | GA |
| ID283 | 19.33333333 | 11.6666667 | 12.33333 | GG |
| ID284 | 17.66666667 | 12.3333333 | 14 | GA |
| ID285 | 18.33333333 | 12.5 | 13.66667 | GA |
| ID286 | 16.33333333 | 10.6666667 | 13 | GG |
| ID287 | 15 | 2 | 11.33333 | GA |
| ID288 | 18 | 11.3333333 | 13.33333 | GG |
| ID289 | 15 | 3.16666667 | 13.33333 | GA |
| ID290 | 18.33333333 | 8.66666667 | 8.666667 | GG |
| ID291 | 16.33333333 | 9.33333333 | 11 | AA |
| ID292 | 17.66666667 | 9.66666667 | 11.33333 | GG |
| ID293 | 16 | 10.3333333 | 13.66667 | GG |
| ID294 | 19.66666667 | 10 | 12 | GG |
| ID295 | 16.33333333 | 10 | 13 | AA |
| ID296 | 18 | 10.3333333 | 12 | GG |
| ID297 | 19.66666667 | 12 | 13.33333 | GG |
| ID298 | 19.33333333 | 12.5 | 15.33333 | GG |
| ID299 | 18.66666667 | 3.33333333 | 11.33333 | GG |
| ID300 | 16.66666667 | 8.5 | 12 | GA |
| ID301 | 17 | 8.33333333 | 11 | GA |
| ID302 | 14.66666667 | 3 | 4.333333 | GG |
| ID303 | 14 | 7.66666667 | 9 | AA |
| ID304 | 16 | 10.6666667 | 12.66667 | AA |
| ID305 | 19.33333333 | 10.6666667 | 10.66667 | GG |
| ID306 | 16 | 2.66666667 | 3.666667 | AA |
| ID307 | 13.66666667 | 8 | 11.66667 | GA |
| ID308 | 16.66666667 | 8.66666667 | 10 | GA |
| ID309 | 16.66666667 | 6 | 7.666667 | AA |
| ID310 | 11.33333333 | 8.33333333 | 13.66667 | AA |
| ID311 | 13.33333333 | 7.66666667 | 10.33333 | GA |
| ID312 | 17.33333333 | 6.66666667 | 8.666667 | GA |
| ID313 | 17.66666667 | 8.33333333 | 10 | AA |
| ID314 | 20 | 10.6666667 | 9.333333 | GG |
| ID315 | 18 | 8.33333333 | 7.666667 | GG |
| ID316 | 15.33333333 | 3 | 11.33333 | AA |
| ID317 | 19.33333333 | 8.66666667 | 7.333333 | GG |
| ID318 | 16.33333333 | 4 | 2.666667 | GA |
| ID319 | 15.33333333 | 8 | 8.666667 | GA |
| ID320 | 15 | 3.5 | 14 | GA |
| ID321 | 15 | 7.16666667 | 10 | AA |
| ID322 | 15 | 8 | 8.666667 | GA |
| ID323 | 18.66666667 | 8.66666667 | 8.666667 | GG |
| ID324 | 15 | 8.16666667 | 9.666667 | AA |
| ID325 | 17.33333333 | 9.83333333 | 12 | GA |
| ID326 | 17.66666667 | 9.5 | 10 | AA |
| ID327 | 14.33333333 | 10.6666667 | 14.66667 | GA |
| ID328 | 18.33333333 | 11.1666667 | 12.33333 | GG |
| ID329 | 17.66666667 | 11.6666667 | 12.33333 | GA |
| ID330 | 18 | 9.5 | 13.33333 | GG |
| ID331 | 18 | 8 | 8 | GG |
| ID332 | 16.66666667 | 12 | 14.33333 | GA |
| ID333 | 14 | 12.3333333 | 14.33333 | AA |
| ID334 | 20 | 6.66666667 | 4.666667 | GG |
| ID335 | 17 | 7 | 7.333333 | GA |
| ID336 | 16.33333333 | 9.33333333 | 8.666667 | GA |
| ID337 | 13.33333333 | 11.6666667 | 11.33333 | GA |
| ID338 | 16.66666667 | 8 | 9.333333 | AA |
| ID339 | 14 | 7.16666667 | 9.666667 | AA |
| ID340 | 19.66666667 | 10.1666667 | 10.66667 | GG |
| ID341 | 16 | 5 | 6.666667 | GA |
| ID342 | 17.33333333 | 8 | 6.666667 | GA |
| ID343 | 16 | 1.83333333 | 3.666667 | GA |
| ID344 | 19 | 12.3333333 | 14.33333 | GG |
| ID345 | 17 | 8 | 10 | GG |
| ID346 | 16.33333333 | 5 | 11 | AA |
| ID347 | 16.66666667 | 10.6666667 | 13.66667 | GA |
| ID348 | 15.33333333 | 7 | 8 | GA |
| ID349 | 15 | 8.66666667 | 10 | AA |
| ID350 | 16 | 6.66666667 | 7.333333 | GA |
| ID351 | 17.66666667 | 10.6666667 | 11 | GA |
| ID352 | 15.66666667 | 9 | 11.33333 | GA |
| ID353 | 15.33333333 | 8 | 8.333333 | GA |
| ID354 | 15.33333333 | 8.66666667 | 12.33333 | GG |
| ID355 | 16.33333333 | 10 | 12 | GG |
| ID356 | 18.33333333 | 11.6666667 | 13.66667 | GG |
| ID357 | 16 | 11.6666667 | 14.33333 | GA |
| ID358 | 16.66666667 | 1.66666667 | 3.333333 | GA |
| ID359 | 15.66666667 | 11 | 13 | GA |
| ID360 | 13.66666667 | 7.66666667 | 9.333333 | GA |
| ID361 | 15 | 9 | 9.333333 | GG |
| ID362 | 19.33333333 | 7 | 4.666667 | GG |
| ID363 | 18 | 13.3333333 | 13 | GG |
| ID364 | 16.33333333 | 9 | 9.666667 | GA |
| ID365 | 16.33333333 | 9.5 | 11.66667 | GA |
| ID366 | 17 | 9.16666667 | 7 | GA |
| ID367 | 18 | 1.16666667 | 2.333333 | GG |
| ID368 | 15 | 9.16666667 | 11.66667 | GA |
| ID369 | 17.33333333 | 4 | 10.33333 | AA |
| ID370 | 13.33333333 | 7.5 | 11 | GA |
| ID371 | 15.66666667 | 7 | 9 | GA |
| ID372 | 16.66666667 | 2.66666667 | 4 | GA |
| ID373 | 17.33333333 | 9 | 10.33333 | GA |
| ID374 | 18.33333333 | 10.1666667 | 10 | GG |
| ID375 | 17 | 11.3333333 | 15.33333 | GG |
| ID376 | 19.33333333 | 10.6666667 | 12 | GG |
| ID377 | 14.33333333 | 6.66666667 | 8.333333 | GG |
| ID378 | 12 | 11 | 13.66667 | GG |
| ID379 | 19 | 10.3333333 | 10 | GG |
| ID380 | 15.66666667 | 8.66666667 | 9.666667 | GG |
| ID381 | 17.66666667 | 9.33333333 | 10.66667 | GG |
| ID382 | 14.66666667 | 7.83333333 | 7.666667 | GA |
| ID383 | 14.66666667 | 10.6666667 | 14.66667 | GA |
| ID384 | 18 | 12 | 11.66667 | GG |
| ID385 | 18.66666667 | 4 | 3.333333 | GA |
| ID386 | 16.33333333 | 8 | 10 | GA |
| ID387 | 15.33333333 | 8.66666667 | 12.33333 | GA |
| ID388 | 18.66666667 | 13.6666667 | 15 | GA |
| ID389 | 14.33333333 | 9 | 13.33333 | GA |
| ID390 | 16.33333333 | 10.6666667 | 12 | GA |
| ID391 | 13.66666667 | 4.33333333 | 6 | GA |
| ID392 | 17.66666667 | 7.33333333 | 8 | GA |
| ID393 | 16 | 6 | 6.666667 | AA |
| ID394 | 16 | 8.83333333 | 13 | GA |
| ID395 | 17 | 7.33333333 | 9 | GA |
| ID396 | 16.66666667 | 9.33333333 | 8 | GA |
| ID397 | 17 | 6.66666667 | 6.333333 | AA |
| ID398 | 17 | 9 | 11 | GA |
| ID399 | 17.66666667 | 10.3333333 | 9.666667 | GA |
| ID400 | 19.33333333 | 11.3333333 | 10.66667 | GA |
| ID401 | 18.33333333 | 9 | 10 | GA |
| ID402 | 17.66666667 | 10.6666667 | 11.33333 | GA |
| ID403 | 18 | 10 | 11.66667 | GA |
| ID404 | 15.33333333 | 2.33333333 | 4 | GA |
| ID405 | 12.66666667 | 9 | 13 | GA |
| ID406 | 19.33333333 | 7.33333333 | 7.333333 | GA |
| ID407 | 18 | 13.3333333 | 14 | GA |
| ID408 | 16 | 7.66666667 | 10 | GA |
| ID409 | 17 | 9.66666667 | 10.66667 | GA |
| ID410 | 16.33333333 | 9.33333333 | 10.66667 | GA |
| ID411 | 15.66666667 | 10 | 12 | GA |
| ID412 | 17 | 9 | 11 | AA |
| ID413 | 16.66666667 | 9.66666667 | 12.66667 | GA |
| ID414 | 19.66666667 | 4 | 12.33333 | AA |
| ID415 | 16.33333333 | 3 | 3.333333 | GA |
| ID416 | 13 | 2.33333333 | 3.333333 | GA |
| ID417 | 15.66666667 | 12.6666667 | 14 | GA |
| ID418 | 18 | 10.1666667 | 10.33333 | GA |
| ID419 | 18 | 12.3333333 | 12.66667 | GA |
| ID420 | 15.66666667 | 9 | 7 | GA |
| ID421 | 0 | 0 | 0 | AA |
| ID422 | 18 | 10.1666667 | 13.66667 | GA |
| ID423 | 15.33333333 | 6.33333333 | 8 | GA |
| ID424 | 16.33333333 | 6.33333333 | 8.666667 | GA |
| ID425 | 18 | 10.1666667 | 13.66667 | GG |
| ID426 | 15.33333333 | 4.66666667 | 6 | GA |
| ID427 | 13.66666667 | 9.33333333 | 10.66667 | GA |
| ID428 | 17 | 2.66666667 | 2.333333 | GA |
| ID429 | 14 | 7.66666667 | 11.66667 | GA |
| ID430 | 0 | 0 | 0 | AA |
| ID431 | 18 | 9.33333333 | 10 | GA |
| ID432 | 17.33333333 | 10.6666667 | 12.66667 | GA |
| ID433 | 17.33333333 | 5.33333333 | 7.333333 | GA |
| ID434 | 17 | 9 | 8.333333 | GA |
| ID435 | 18.66666667 | 5.33333333 | 2.666667 | GA |
| ID436 | 13.33333333 | 7 | 9.333333 | AA |
| ID437 | 14.33333333 | 8.66666667 | 9 | GA |
| ID438 | 10 | 2.66666667 | 4 | GA |
| ID439 | 20 | 12.8333333 | 13 | GA |
| ID440 | 18.66666667 | 13 | 14.33333 | GA |
| ID441 | 13.66666667 | 9.66666667 | 14 | GA |
| ID442 | 14.33333333 | 3 | 3.333333 | GA |
| ID443 | 15.66666667 | 9.66666667 | 13 | GA |
| ID444 | 17.33333333 | 12 | 12.33333 | GA |
| ID445 | 15.33333333 | 10 | 14 | GA |
| ID446 | 16.66666667 | 6.66666667 | 4 | GA |
| ID447 | 17.33333333 | 8.5 | 10 | GA |
| ID448 | 13.33333333 | 8.66666667 | 12.33333 | GA |
| ID449 | 15.33333333 | 2.16666667 | 3.333333 | GA |
| ID450 | 17.33333333 | 9.66666667 | 10 | GA |
| ID451 | 15.33333333 | 7 | 8.666667 | GA |
| ID452 | 17.66666667 | 11.6666667 | 13.33333 | GA |
| ID453 | 19.66666667 | 12.6666667 | 12.33333 | GA |
| ID454 | 17.66666667 | 8 | 8.666667 | GA |
| ID455 | 14.33333333 | 8.33333333 | 12.33333 | GA |
| ID456 | 16.33333333 | 10.6666667 | 12.66667 | GA |
| ID457 | 16.33333333 | 9.66666667 | 11.66667 | GA |
| ID458 | 15.33333333 | 5.33333333 | 4 | GG |
| ID459 | 16 | 9.5 | 11.66667 | GA |
| ID460 | 16 | 8.83333333 | 11 | GG |
| ID461 | 17 | 12.3333333 | 16.66667 | GA |
| ID462 | 15.66666667 | 10 | 12.66667 | GA |
| ID463 | 19.66666667 | 11.3333333 | 11 | GA |
| ID464 | 13 | 1.66666667 | 2.333333 | GG |
| ID465 | 16.66666667 | 12.3333333 | 14.33333 | GA |
| ID466 | 15 | 1.33333333 | 2 | GA |
| ID467 | 16 | 3.33333333 | 4 | GA |
| ID468 | 14.33333333 | 9.33333333 | 14 | GA |
| ID469 | 18.33333333 | 10.8333333 | 11.66667 | GG |
| ID470 | 15 | 7.66666667 | 9.666667 | GG |
| ID471 | 15.33333333 | 11.3333333 | 12.66667 | GA |
| ID472 | 19 | 13.3333333 | 15 | GA |
| ID473 | 13.33333333 | 9.5 | 13.66667 | GA |
| ID474 | 16 | 8.66666667 | 10 | GG |
| ID475 | 17.33333333 | 12 | 14.33333 | GG |
| ID476 | 15.33333333 | 10.3333333 | 11.66667 | GG |
| ID477 | 15.66666667 | 8.33333333 | 9.666667 | GG |
| ID478 | 16 | 7.83333333 | 9 | GG |
| ID479 | 16.33333333 | 11.8333333 | 12.66667 | GG |
| ID480 | 17.66666667 | 11.6666667 | 10.33333 | GG |
| ID481 | 17.33333333 | 11 | 12 | GA |
| ID482 | 15.33333333 | 9.66666667 | 13.66667 | GA |
| ID483 | 16.33333333 | 7.33333333 | 7.333333 | AA |
| ID484 | 14.66666667 | 8.16666667 | 11.66667 | AA |
| ID485 | 15.33333333 | 2.5 | 2.666667 | GA |
| ID486 | 16.33333333 | 10.3333333 | 11.66667 | GA |
| ID487 | 0 | 0 | 0 | AA |
| ID488 | 16.33333333 | 11.3333333 | 12 | GA |
| ID489 | 17.66666667 | 9 | 9.666667 | GA |
| ID490 | 17 | 10.3333333 | 11.66667 | GA |
| ID491 | 18.33333333 | 8.33333333 | 10 | GA |
| ID492 | 14.66666667 | 7.33333333 | 10.66667 | GA |
| ID493 | 18.66666667 | 12.5 | 12.33333 | GA |
| ID494 | 14.66666667 | 3.33333333 | 4 | AA |
| ID495 | 18.33333333 | 5 | 11.66667 | AA |
| ID496 | 10.66666667 | 4 | 6.666667 | GA |
| ID497 | 14.66666667 | 4.66666667 | 4 | GA |
| ID498 | 18.33333333 | 12.3333333 | 14 | GA |
| ID499 | 17.66666667 | 10 | 9.333333 | GA |
| ID500 | 16 | 10.6666667 | 13.33333 | AA |
| ID501 | 17.33333333 | 7.66666667 | 9.333333 | GA |
| ID502 | 15.33333333 | 9.66666667 | 13.66667 | GA |
| ID503 | 14 | 10.6666667 | 8.333333 | GA |
| ID504 | 0.5 | 0 | 0 | GA |
| ID505 | 1 | 0 | 0 | GA |
| ID506 | 15.66666667 | 14 | 12.66667 | GA |
| ID507 | 15.33333333 | 14.3333333 | 14 | AA |
| ID508 | 15 | 11.6666667 | 9.666667 | AA |
| ID509 | 14.66666667 | 12 | 12 | GA |
| ID510 | 13.33333333 | 13.6666667 | 15.66667 | GA |
| ID511 | 14.66666667 | 13.3333333 | 12.33333 | AA |
| ID512 | 15.33333333 | 13.3333333 | 12.33333 | GA |
| ID513 | 11.66666667 | 11 | 11.66667 | GA |
| ID514 | 16.33333333 | 12.3333333 | 11 | GA |
| ID515 | 13.66666667 | 12 | 12 | GA |
| ID516 | 15.33333333 | 12 | 9.333333 | GA |
| ID517 | 16 | 2.33333333 | 1.666667 | GA |
| ID518 | 16.66666667 | 7 | 12.33333 | AA |
| ID519 | 14.33333333 | 14.3333333 | 15.66667 | AA |
| ID520 | 14.66666667 | 12.5 | 11.33333 | GA |
| ID521 | 14 | 8 | 7.333333 | GA |
| ID522 | 15 | 8.66666667 | 8.666667 | GA |
| ID523 | 13.66666667 | 11.6666667 | 13.33333 | GA |
| ID524 | 0 | 0 | 0 | AA |
| ID525 | 18 | 10.1666667 | 13.66667 | GA |
| ID526 | 14 | 9.66666667 | 8.666667 | GA |
| ID527 | 15 | 10.3333333 | 9.666667 | GA |
| ID528 | 18 | 10.1666667 | 13.66667 | GA |
| ID529 | 18 | 10.1666667 | 13.66667 | GA |
| ID530 | 15.33333333 | 7.66666667 | 7.333333 | GA |
| ID531 | 13.33333333 | 8.33333333 | 10.66667 | GA |
| ID532 | 12.33333333 | 7.83333333 | 8.333333 | GA |
| ID533 | 10.33333333 | 10 | 9.333333 | GA |
| ID534 | 13 | 7.33333333 | 7.333333 | GA |
| ID535 | 11.33333333 | 7.33333333 | 9.333333 | GA |
| ID536 | 13.33333333 | 8.5 | 9.333333 | AA |
| ID537 | 13 | 7.33333333 | 6 | GA |
| ID538 | 0.5 | 0 | 0 | GA |
| ID539 | 13 | 13 | 12.33333 | GA |
| ID540 | 13.33333333 | 8.5 | 9.333333 | AA |
| ID541 | 13.33333333 | 8.66666667 | 8.666667 | GA |
| ID542 | 15.66666667 | 12.1666667 | 11.33333 | GA |
| ID543 | 11.66666667 | 9 | 8.666667 | GA |
| ID544 | 6.333333333 | 0 | 0 | GA |
| ID545 | 0.333333333 | 0 | 0 | GA |
| ID546 | 12 | 8 | 8.666667 | GA |
| ID547 | 13.33333333 | 8.33333333 | 10.66667 | GA |
| ID548 | 10.33333333 | 0 | 0 | AA |
| ID549 | 11 | 9.33333333 | 8.666667 | GA |
| ID550 | 4.333333333 | 6 | 6 | AA |
| ID551 | 15.66666667 | 15.6666667 | 9.666667 | GA |
| ID552 | 8.666666667 | 0 | 0 | GA |
| ID553 | 0 | 0 | 0 | AA |
| ID554 | 0 | 0 | 0 | AA |
| ID555 | 16 | 9 | 8.666667 | GA |
| ID556 | 11.33333333 | 0 | 0 | GA |
| ID557 | 13.33333333 | 8.33333333 | 10.66667 | GA |
| ID558 | 14.33333333 | 10.6666667 | 10 | AA |
| ID559 | 13.66666667 | 10 | 10 | GA |
| ID560 | 13.33333333 | 12 | 12.33333 | GA |
| ID561 | 16.66666667 | 11 | 10.33333 | GA |
| ID562 | 14.33333333 | 9.66666667 | 10.66667 | GA |
| ID563 | 0 | 0 | 0 | GA |
| ID564 | 14 | 9.33333333 | 9.666667 | GA |
| ID565 | 12.66666667 | 8.33333333 | 8.333333 | GA |
| ID566 | 14 | 9.33333333 | 9 | GA |
| ID567 | 12.66666667 | 8.33333333 | 8.333333 | GA |
| ID568 | 11 | 10.6666667 | 9.333333 | GA |
| ID569 | 14.66666667 | 9.33333333 | 10 | GA |
| ID570 | 14.33333333 | 11.6666667 | 11.33333 | GA |
| ID571 | 11.66666667 | 10.1666667 | 9.333333 | GA |
| ID572 | 15.66666667 | 13.3333333 | 12 | GA |
| ID573 | 16.33333333 | 12.3333333 | 12.66667 | GG |
| ID574 | 9.666666667 | 10 | 10 | GA |
| ID575 | 15.33333333 | 10.6666667 | 7.333333 | GG |
| ID576 | 15.66666667 | 10 | 10 | GG |
| ID577 | 15.33333333 | 14.5 | 15 | GA |
| ID578 | 9 | 8.83333333 | 9.333333 | GA |
| ID579 | 12.66666667 | 8.33333333 | 8.333333 | GA |
| ID580 | 11 | 11 | 12.66667 | GA |
| ID581 | 9.333333333 | 6.66666667 | 6.666667 | GA |
| ID582 | 6.333333333 | 6.66666667 | 7.333333 | GA |
| ID583 | 17 | 9.33333333 | 10.66667 | GA |
| ID584 | 15.33333333 | 7.66666667 | 6.666667 | GA |
| ID585 | 16 | 14 | 11 | GA |
| ID586 | 9.666666667 | 6.66666667 | 6 | GA |
| ID587 | 14.66666667 | 6.66666667 | 6.666667 | GA |
| ID588 | 0.333333333 | 0 | 0 | GA |
| ID589 | 12.33333333 | 6.5 | 6.666667 | GA |
| ID590 | 13.66666667 | 10.3333333 | 11.33333 | GA |
| ID591 | 15.33333333 | 11 | 8.666667 | GA |
| ID592 | 15.33333333 | 11.5 | 11.66667 | AA |
| ID593 | 10 | 7 | 8 | GA |
| ID594 | 15.33333333 | 10.6666667 | 10 | GA |
| ID595 | 11.66666667 | 12.1666667 | 11.66667 | GA |
| ID596 | 16 | 10.6666667 | 10.66667 | GA |
| ID597 | 13.33333333 | 7.33333333 | 7.333333 | AA |
| ID598 | 14 | 9.33333333 | 8.333333 | GA |
| ID599 | 16 | 8.66666667 | 6.666667 | GA |
| ID600 | 12.66666667 | 9.5 | 11 | GA |
| ID601 | 14.66666667 | 6 | 11 | AA |
| ID602 | 15 | 10.6666667 | 9.666667 | GA |
| ID603 | 13 | 10.6666667 | 11 | GA |
| ID604 | 14.33333333 | 9.66666667 | 8.666667 | AA |
| ID605 | 4.666666667 | 6 | 6 | GA |
| ID606 | 15.33333333 | 11 | 12 | AA |
| ID607 | 16 | 10.6666667 | 9.333333 | GA |
| ID608 | 16 | 12.3333333 | 10 | GA |
| ID609 | 14 | 11.3333333 | 13.33333 | GA |
| ID610 | 12.66666667 | 8.33333333 | 8.333333 | GA |
| ID611 | 12.66666667 | 7.83333333 | 6.666667 | GA |
| ID612 | 12.66666667 | 8.33333333 | 8.333333 | GA |
| ID613 | 12 | 11.3333333 | 10.33333 | GA |
| ID614 | 15 | 9.16666667 | 7.333333 | GA |
| ID615 | 14.33333333 | 10.3333333 | 9 | GA |
| ID616 | 12.33333333 | 10 | 11 | GA |
| ID617 | 14.66666667 | 8.33333333 | 6.666667 | GA |
| ID618 | 9.666666667 | 6.66666667 | 6.666667 | GA |
| ID619 | 15.66666667 | 12.3333333 | 13.33333 | GA |
| ID620 | 13.66666667 | 7.33333333 | 6 | GA |
| ID621 | 15 | 7.33333333 | 8 | GA |
| ID622 | 12.66666667 | 7.33333333 | 7.333333 | GA |
| ID623 | 14 | 9.66666667 | 10.33333 | GA |
| ID624 | 14 | 10.6666667 | 10 | GA |
| ID625 | 1 | 7.33333333 | 6 | GA |
| ID626 | 12.66666667 | 8.33333333 | 8.333333 | GA |
| ID627 | 8 | 8.66666667 | 11 | AA |
| ID628 | 14.66666667 | 10.6666667 | 10.66667 | GA |
| ID629 | 13.66666667 | 9.33333333 | 8 | GA |
| ID630 | 15 | 10.6666667 | 10 | GA |
| ID631 | 14.66666667 | 9.16666667 | 9 | GA |
| ID632 | 15.66666667 | 10.6666667 | 12.33333 | GA |
| ID633 | 14.66666667 | 12.3333333 | 14 | GA |
| ID634 | 13 | 10.6666667 | 10.33333 | GA |
| ID635 | 14.66666667 | 12.3333333 | 14 | GA |
| ID636 | 12.66666667 | 8.33333333 | 8.333333 | GA |
| ID637 | 14.33333333 | 10 | 9.333333 | GA |
| ID638 | 11 | 9 | 8.666667 | GA |
| ID639 | 12.66666667 | 8.66666667 | 7.333333 | AA |
| ID640 | 14 | 8 | 7.333333 | GA |
| ID641 | 13.66666667 | 11 | 11.66667 | GA |
| ID642 | 15.33333333 | 10.3333333 | 9.666667 | GA |
| ID643 | 3 | 7.66666667 | 8.666667 | AA |
| ID644 | 14.33333333 | 8.66666667 | 9 | GA |
| ID645 | 5.333333333 | 0.5 | 2.333333 | GA |
| ID646 | 13.66666667 | 10 | 9 | AA |
| ID647 | 13.66666667 | 8.5 | 7.333333 | GA |
| ID648 | 12.66666667 | 10 | 9 | GA |
| ID649 | 15 | 8.33333333 | 7 | GA |
| ID650 | 14.33333333 | 8 | 14 | AA |
| ID651 | 13 | 8 | 8.333333 | AA |
| ID652 | 14.66666667 | 7 | 7 | GA |
| ID653 | 3 | 6.66666667 | 6.666667 | GA |
| ID654 | 15 | 8.33333333 | 8 | GA |
| ID655 | 5.333333333 | 7.16666667 | 9.666667 | GA |
| ID656 | 13.33333333 | 9 | 9.333333 | GA |
| ID657 | 1 | 0.33333333 | 3 | GA |
| ID658 | 10 | 2.66666667 | 4 | GA |
| ID659 | 12.33333333 | 6 | 9.333333 | GA |
| ID660 | 12.66666667 | 7.5 | 11.33333 | GA |
| ID661 | 17 | 4.66666667 | 7.333333 | GA |
| ID662 | 18 | 9 | 13.33333 | AA |
| ID663 | 18 | 9.33333333 | 10 | GA |
| ID664 | 17.33333333 | 2 | 3 | AA |
| ID665 | 18.66666667 | 11.3333333 | 13 | GA |
| ID666 | 11.33333333 | 1.66666667 | 4 | GA |
| ID667 | 15 | 7.66666667 | 11.66667 | GA |
| ID668 | 17.33333333 | 10.3333333 | 13.33333 | GA |
| ID669 | 13.66666667 | 2.66666667 | 3.333333 | GA |
| ID670 | 18.66666667 | 11.3333333 | 12.66667 | GA |
| ID671 | 16.66666667 | 9.83333333 | 11.33333 | GA |
| ID672 | 19.33333333 | 5 | 6.666667 | GA |
| ID673 | 19.33333333 | 6 | 5.333333 | AA |
| ID674 | 16.33333333 | 9.16666667 | 9.666667 | GA |
| ID675 | 16.66666667 | 5.33333333 | 5.333333 | GA |
| ID676 | 16.66666667 | 3 | 5.333333 | GA |
| ID677 | 15 | 3.16666667 | 13.33333 | GA |
| ID678 | 15.33333333 | 11.3333333 | 15 | GA |
| ID679 | 14 | 2.33333333 | 13 | GA |
| ID680 | 10.33333333 | 5.66666667 | 12 | AA |
| ID681 | 15.33333333 | 2 | 3.333333 | GA |
| ID682 | 9 | 1.83333333 | 3.333333 | GA |
| ID683 | 13.66666667 | 0 | 0 | GA |
| ID684 | 14.66666667 | 11.3333333 | 12 | GA |
| ID685 | 13.33333333 | 10.6666667 | 11.33333 | GA |
| ID686 | 14 | 11.6666667 | 12 | GA |
| ID687 | 15 | 16 | 9.666667 | AA |
| ID688 | 16 | 14.3333333 | 13.66667 | GA |
| ID689 | 15.33333333 | 11.6666667 | 11.33333 | AA |
| ID690 | 16.66666667 | 14.6666667 | 13.66667 | AA |
| ID691 | 14 | 14 | 15 | GA |
| ID692 | 14.66666667 | 12 | 12 | GA |
| ID693 | 13.33333333 | 9.33333333 | 10 | GA |
| ID694 | 15 | 11 | 7.666667 | GA |
| ID695 | 10.33333333 | 8.33333333 | 6.666667 | GA |
| ID696 | 14.33333333 | 6.5 | 12.66667 | GA |
| ID697 | 16 | 11.1666667 | 12.66667 | GA |
| ID698 | 10 | 9.33333333 | 10 | GA |
| ID699 | 13.66666667 | 5.66666667 | 8 | GA |
| ID700 | 11.66666667 | 5 | 10 | GA |
| ID701 | 5.666666667 | 1.33333333 | 0 | AA |
| ID702 | 12.33333333 | 7.33333333 | 9.333333 | AA |
| ID703 | 17 | 4.33333333 | 8 | GA |
| ID704 | 8.333333333 | 7.33333333 | 8.666667 | GA |
| ID705 | 19 | 12 | 12.66667 | GA |
| ID706 | 17.33333333 | 8.66666667 | 8.666667 | GA |
| ID707 | 15.66666667 | 9 | 11 | GA |
| ID708 | 18.33333333 | 4.66666667 | 5.333333 | AA |
| ID709 | 18 | 9 | 6.666667 | GA |
| ID710 | 10 | 4.33333333 | 7.666667 | AA |
| ID711 | 16.33333333 | 6.5 | 10.33333 | GA |
| ID712 | 13 | 5 | 9 | GA |
| ID713 | 16 | 8 | 8.666667 | GA |
| ID714 | 19.66666667 | 11.3333333 | 10 | GG |
| ID715 | 15.66666667 | 7.66666667 | 10.33333 | GA |
| ID716 | 14.66666667 | 8.33333333 | 10.33333 | GG |
| ID717 | 14.66666667 | 5.66666667 | 7 | GA |
| ID718 | 15.66666667 | 10 | 10 | GG |
| ID719 | 14.33333333 | 1.66666667 | 2.666667 | GA |
| ID720 | 15 | 8.66666667 | 10 | GA |
| ID721 | 15 | 9 | 12.66667 | GA |
| ID722 | 17.66666667 | 9 | 11.33333 | GA |
| ID723 | 7.666666667 | 3.33333333 | 9 | AA |
| ID724 | 16.66666667 | 5 | 11.66667 | AA |
| ID725 | 0 | 0 | 0 | AA |
| ID726 | 17 | 9 | 11.33333 | GA |
| ID727 | 14 | 10.8333333 | 13.33333 | GA |
| ID728 | 13 | 3 | 4.333333 | GA |
| ID729 | 8.333333333 | 5.33333333 | 8 | GA |
| ID730 | 19.66666667 | 7.66666667 | 8 | GA |
| ID731 | 15.33333333 | 1.33333333 | 12.33333 | GA |
| ID732 | 17.66666667 | 6.66666667 | 6 | GA |
| ID733 | 14.33333333 | 7.66666667 | 10.33333 | GA |
| ID734 | 17.33333333 | 10.6666667 | 12 | GA |
| ID735 | 15 | 8 | 10.33333 | GA |
| ID736 | 13.33333333 | 2.33333333 | 3.333333 | GA |
| ID737 | 3.666666667 | 4 | 4.666667 | GA |
| ID738 | 8 | 0 | 0 | GA |
| ID739 | 18.33333333 | 10.3333333 | 11.33333 | GA |
| ID740 | 6.333333333 | 0 | 6.666667 | GA |
| ID741 | 11 | 8.66666667 | 10 | GA |
| ID742 | 14.66666667 | 2.66666667 | 2.333333 | GG |
| ID743 | 9 | 4.66666667 | 6.666667 | GA |
| ID744 | 15.33333333 | 11 | 11.66667 | GG |
| ID745 | 17.66666667 | 9 | 7.333333 | GA |
| ID746 | 1.666666667 | 0 | 0 | GA |
| ID747 | 16.33333333 | 8 | 10 | GA |
| ID748 | 13.33333333 | 8.33333333 | 10.66667 | GA |
| ID749 | 16 | 12 | 14 | GA |
| ID750 | 17 | 2 | 2 | GA |
| ID751 | 10.33333333 | 0 | 0 | AA |
| ID752 | 14.66666667 | 10 | 12.66667 | GA |
| ID753 | 7.666666667 | 3.66666667 | 7.333333 | GA |
| ID754 | 15 | 6 | 6 | GA |
| ID755 | 17.66666667 | 1.66666667 | 2.666667 | GA |
| ID756 | 17 | 3.33333333 | 5.333333 | GA |
| ID757 | 15.33333333 | 2 | 2.666667 | GA |
| ID758 | 12 | 5.33333333 | 7 | GA |
| ID759 | 17.33333333 | 6.33333333 | 6 | GA |
| ID760 | 0.666666667 | 0 | 0 | GA |
| ID761 | 19.33333333 | 9 | 6 | GA |
| ID762 | 16.66666667 | 2.5 | 4 | AA |
| ID763 | 2 | 0 | 0 | GA |
| ID764 | 17.66666667 | 9.33333333 | 11 | GA |
| ID765 | 15.66666667 | 9 | 9.666667 | GA |
| ID766 | 16 | 2.33333333 | 9.666667 | GA |
| ID767 | 18.33333333 | 11.1666667 | 13.66667 | GA |
| ID768 | 18 | 9.66666667 | 9.333333 | GA |
| ID769 | 16 | 2.33333333 | 1.666667 | AA |
| ID770 | 11.33333333 | 1.66666667 | 4 | AA |
| ID771 | 10 | 9.33333333 | 10 | GG |
| ID772 | 14.66666667 | 7.66666667 | 7 | GG |
| ID773 | 17 | 9 | 13 | AA |
| ID774 | 16 | 10 | 12 | GA |
| ID775 | 15.66666667 | 5 | 11.33333 | AA |
| ID776 | 18 | 11.3333333 | 10 | GA |
| ID777 | 17.33333333 | 3 | 4.333333 | GA |
| ID778 | 18.33333333 | 11.1666667 | 13.66667 | GG |
| ID779 | 18.66666667 | 12.3333333 | 13.66667 | GG |
| ID780 | 17.33333333 | 12 | 14.66667 | GA |
| ID781 | 17.66666667 | 11 | 11 | GG |
| ID782 | 16.33333333 | 6 | 7.333333 | GA |
| ID783 | 15.66666667 | 6.66666667 | 6.666667 | GG |
| ID784 | 11 | 3.66666667 | 4.666667 | GG |
| ID785 | 15.33333333 | 7.33333333 | 11.66667 | GG |
| ID786 | 16.33333333 | 10.3333333 | 9.666667 | GG |
| ID787 | 18.33333333 | 11.3333333 | 7 | GG |
| ID788 | 17.66666667 | 10.6666667 | 9.666667 | GA |
| ID789 | 16.33333333 | 10 | 13 | GA |
| ID790 | 14.33333333 | 9.66666667 | 14.33333 | GA |
| ID791 | 17 | 9.83333333 | 11 | GA |
| ID792 | 16.33333333 | 10.5 | 12 | GA |
| ID793 | 19.66666667 | 13.3333333 | 12.33333 | GG |
| ID794 | 18 | 11.8333333 | 14.33333 | GA |
| ID795 | 16.33333333 | 9.66666667 | 9.666667 | GA |
| ID796 | 13.33333333 | 3.66666667 | 6 | GA |
| ID797 | 17.33333333 | 11.5 | 14 | GA |
| ID798 | 16.66666667 | 11.6666667 | 13 | GA |
| ID799 | 15.66666667 | 7 | 10.33333 | GA |
| ID800 | 15.66666667 | 7.83333333 | 9.666667 | GA |
| ID801 | 15 | 6.66666667 | 8 | GA |
| ID802 | 14.66666667 | 8.33333333 | 11.66667 | GA |
| ID803 | 15.33333333 | 1.66666667 | 3 | GA |
| ID804 | 18.66666667 | 2.33333333 | 10.33333 | GA |
| ID805 | 18 | 13.6666667 | 14.66667 | GA |
| ID806 | 18.33333333 | 11.6666667 | 10 | AA |
| ID807 | 17.66666667 | 8.66666667 | 6.333333 | GA |
| ID808 | 17.66666667 | 10 | 10 | GA |
| ID809 | 19.66666667 | 11.3333333 | 8.333333 | GA |
| ID810 | 18 | 12.3333333 | 13 | GA |
| ID811 | 15.33333333 | 9.16666667 | 7.666667 | GA |
| ID812 | 17.33333333 | 9 | 11 | GA |
| ID813 | 13.33333333 | 9.33333333 | 13 | GA |
| ID814 | 15.66666667 | 10 | 10 | GG |
| ID815 | 15.66666667 | 10 | 10 | GG |
| ID816 | 17.33333333 | 9 | 8.666667 | GA |
| ID817 | 17.66666667 | 7.66666667 | 9.333333 | GG |
| ID818 | 17 | 7 | 10 | AA |
| ID819 | 14 | 9 | 8.666667 | GA |
| ID820 | 18 | 2 | 2.666667 | GA |
| ID821 | 14.66666667 | 6 | 9.333333 | AA |
| ID822 | 3.666666667 | 4 | 4.666667 | GA |
| ID823 | 15.33333333 | 5 | 11.66667 | AA |
| ID824 | 17 | 9.16666667 | 7 | GA |
| ID825 | 14.66666667 | 7.83333333 | 7.666667 | GA |
| ID826 | 14 | 4.66666667 | 8 | GA |

**Notes:**; Wg_no (wing number); EN (egg number); FN (the number of fertile eggs after a single AI); DN (the number of days post-insemination until last fertile egg).
